# A suite of eight *Toxoplasma gondii* effectors cooperates to activate the non-canonical NF-κB pathway

**DOI:** 10.1128/mbio.01652-26

**Published:** 2026-08-28

**Authors:** Kenna Berg, Michael W. Panas, Samarchith P. Kurup, John C. Boothroyd, Alex Rosenberg

**Affiliations:** 1 Department of Infectious Diseases, University of Georgia 186969 , Athens, Georgia, USA; 2 Center for Tropical and Emerging Global Diseases, University of Georgia 186969 , Athens, Georgia, USA; 3 Department of Microbiology and Immunology, Stanford University School of Medicine 10624, Stanford, California, USA; 4 Department of Cellular Biology, University of Georgia 186969 , Athens, Georgia, USA; Gulbenkian Institute for Molecular Medicine Oeiras Site, Lisbon, Portugal

**Keywords:** *Toxoplasma gondii*, non-canonical NF-κB, dense granule effectors, MYR translocon, RelB, p52, NIK stabilization, TRAF3

## Abstract

As a master of host-cell reprogramming, *Toxoplasma gondii* (*T. gondii*) tachyzoites manipulate diverse signaling networks to establish a niche permissive for long-term infection. While the parasite’s subversion of canonical NF-κB signaling (p65/p50) is well established, how infection impacts the non-canonical NF-κB pathway has been largely unexplored. Here, we report that *T. gondii* infection induces nuclear accumulation of the non-canonical NF-κB subunits RelB and p52 in both human and murine fibroblasts. This response is conserved across both type I and type II parasite genetic backgrounds. We demonstrate that this reprogramming is dependent on the MYR1-mediated export of dense granule effectors. Mechanistically, *T. gondii* infection drives the depletion of the negative regulator TRAF3, leading to the stabilization of NF-κB-inducing kinase (NIK), phosphorylation of p100, and its subsequent processing into p52. Utilizing a panel of combinatorial knockout parasites, we reveal that no single effector is responsible for this phenotype. Instead, a suite of eight MYR1-dependent effectors, IST, NSM, HCE1/TEEGR, GRA16, GRA18, GRA24, GRA28, and GRA84, functions through a collaborative, additive network to drive most of the non-canonical response. These findings highlight a distributed regulatory strategy used by the parasite to overcome host transcriptional robustness and shape host signaling.

**IMPORTANCE:**

*Toxoplasma gondii* infects nearly one-third of the global population and establishes infection by extensively rewiring host immune signaling. While decades of work have focused on how the parasite modulates canonical NF-κB activity, whether it also engages the alternative, non-canonical arm of this pathway has remained unclear. Here, we show that *T. gondii* tachyzoites activate non-canonical NF-κB signaling, driving nuclear accumulation of RelB/p52 through MYR1-dependent effector export. Unexpectedly, no single effector is responsible. Instead, eight secreted proteins act cooperatively to enable NIK stabilization and engage the non-canonical NF-κB cascade, revealing a networked mode of immune control. This discovery highlights a regulatory logic evolved by the parasite to overcome host transcriptional robustness. Together, these findings identify non-canonical NF-κB activation as a new axis of host-parasite interaction and expand our understanding of how *T. gondii* reprograms central immune signaling circuits through multi-effector networks.

## INTRODUCTION

*Toxoplasma gondii* (*T. gondii*) is an obligate intracellular apicomplexan parasite that infects approximately one-third of the human population (1). It causes severe disease in immunocompromised individuals and congenitally infected infants (2). In North America and Europe, the parasite population is dominated by a small number of clonal lineages: type I strains are acutely virulent, while type II and type III strains show intermediate and low virulence in laboratory mice, respectively (3). A central feature of *T. gondii* pathogenesis is the establishment of the parasitophorous vacuole (PV), a specialized niche that protects the parasite while allowing it to reprogram host cell pathways (4). This remodeling is driven by the delivery of effector proteins from rhoptries and dense granules into the host cell (5).

The transport of exported dense granule effectors across the parasitophorous vacuole membrane into the host cell appears to be mediated by a putative translocon complex thought to include MYR1-MYR4, of which only MYR1 and MYR3 have been shown to stably associate (6–9). This machinery is supported by the ASP5 protease, GRA45 chaperone, ROP17 kinase, GRA44 phosphatase, and the accessory protein GRA59 (10–13). To date, eight MYR1-dependent exported dense granule effectors have been identified, the majority of which localize to the host nucleus: IST, NSM, HCE1/TEEGR, GRA16, GRA18, GRA24, GRA28, and the recently characterized host nuclear effector GRA84 (14–23). MYR1-dependent effectors account for the majority of *T. gondii-*driven host transcriptional reprogramming (24). Our recent work and that of others indicate that *T. gondii* effectors often function in redundant, synergistic, or antagonistic networks rather than as isolated factors. This combinatorial logic is prominent in the subversion of two major host pathways: IFN-γ and NF-κB (24–26).

In the subversion of the IFN-γ signaling pathway, IST blocks STAT1-dependent transcription by recruiting Mi-2/NuRD to STAT1-bound promoters and preventing coactivator engagement, while NSM reinforces repression during chronic infection by elevating NCoR/SMRT repressor activity (19, 21). In parallel, functional genetic studies support a redundant network of four MYR-dependent nuclear effectors, IST, GRA16, GRA24, and GRA28, that collectively suppress IFN-γ-driven programs and prevent IFN-γ-associated host cell death and premature parasite egress (25).

A similar combinatorial logic shapes NF-κB-mediated inflammatory signaling during infection. In type II strains, GRA15 serves as a dominant driver of canonical NF-κB activation by recruiting TRAF2 and TRAF6 to promote IKK activation and p65 nuclear translocation, with additional effectors and feedback mechanisms shaping p65 nuclear accumulation and downstream cytokine output (27–29). GRA24 further modulates this response by contributing to c-Rel nuclear accumulation, similar to GRA15, although neither effector is strictly required (30). Other effectors restrain pathway output to limit pathology, including the nuclear repressor HCE1/TEEGR, which recruits EZH2-linked machinery to silence subsets of NF-κB target genes (15). In infected macrophages, these same NF-κB inputs are repurposed toward dissemination by promoting NF-κB-dependent CCR7 expression. GRA28 acts as a central enhancer by increasing chromatin accessibility at the *Ccr7* locus via SWI/SNF, thereby potentiating GRA15/24-driven, NF-κB-dependent transcription. Together, these activities promote CCR7-dependent hypermigration, with additional support from GRA16 and GRA18 and counter-regulation by HCE1/TEEGR (26, 31).

Despite extensive attention to canonical NF-κB regulation during *T. gondii* infection, whether and how the parasite engages the alternative, non-canonical NF-κB pathway has remained less clear (32). The non-canonical pathway is defined by inducible processing of p100 into p52 and subsequent nuclear accumulation of RelB and p52 complexes, typically downstream of a subset of TNF-receptor family members and related signaling axes (33). Mechanistically, non-canonical activation is governed by the stability of NF-κB-inducing kinase (NIK) (34). Under resting conditions, NIK is kept low through a TRAF2-, TRAF3-, and cIAP-dependent ubiquitination system; pathway activation involves TRAF3 degradation and NIK stabilization, leading to IKKα-driven phosphorylation of p100 and its processing to p52 (35).

Early studies demonstrated that this pathway plays a critical role during *T. gondii* infection. RELB-deficient mice are highly susceptible to infection, succumbing rapidly due to a profound failure in infection-induced activation of NK cells and the production of protective IFN-γ by T cells (36). While these foundational studies underscored the necessity of RelB for both innate and adaptive immunity, the molecular strategies employed by *T. gondii* to interact with this pathway have remained unknown.

Here, we identify activation of the non-canonical NF-κB pathway as a previously unrecognized consequence of *T. gondii* infection in human and mouse fibroblasts infected with type I and type II parasites. Surprisingly, this response does not appear to be explained by any single exported effector but instead emerges from a distributed mode of host cell reprogramming. These findings reveal a new dimension of *Toxoplasma*-host interaction and suggest that parasite engagement of non-canonical NF-κB mirrors the multilayered regulatory logic that governs this pathway in uninfected cells.

## RESULTS

### MYR1-dependent effector export drives nuclear accumulation of RelB and p52

**Fig 1:**
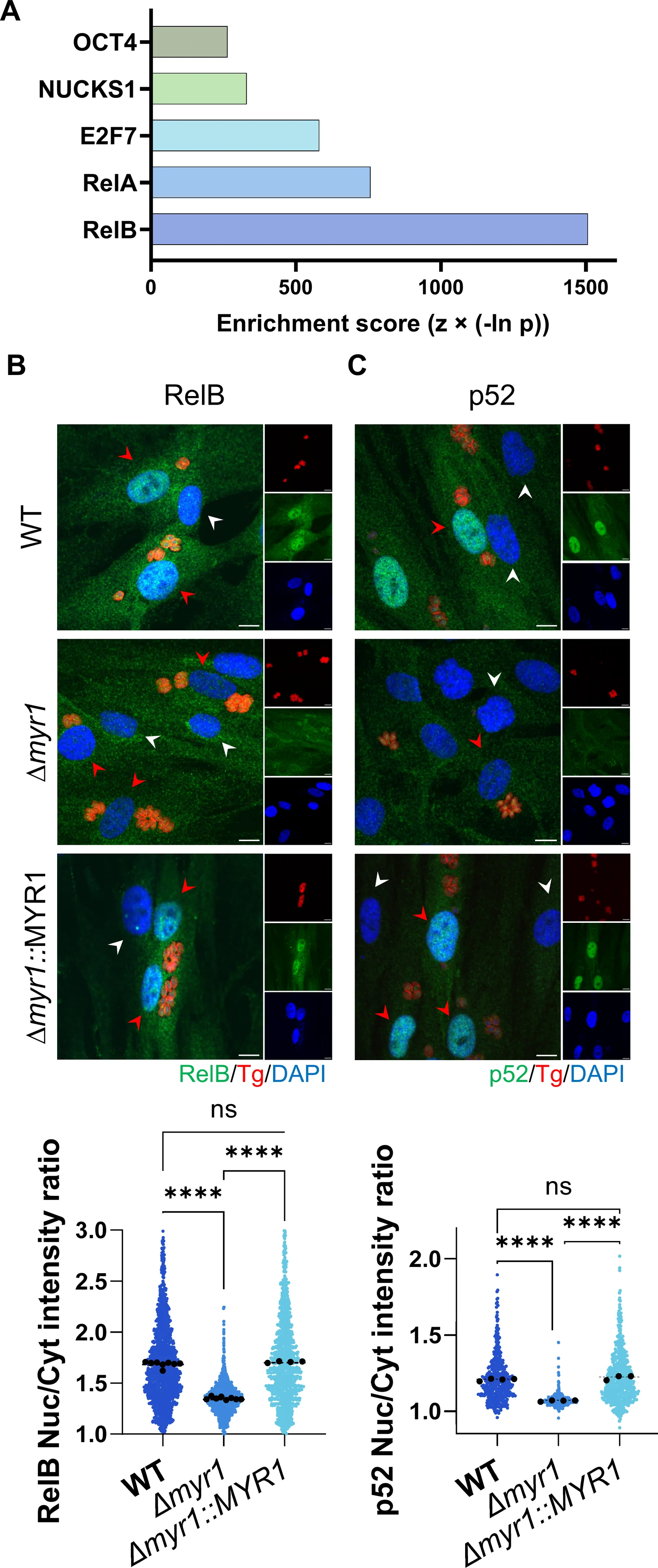
MYR1-dependent effector export drives nuclear accumulation of RelB and p52. (**A**) Top-scoring transcription factors predicted to regulate the MYR1-dependent upregulated genes in human foreskin fibroblast (HFF) cells relative to uninfected controls were identified by analysis of previously published data (24) using Enrichr. (**B and C**) Representative images (top) and quantitative analysis (bottom) for RelB (**B**) and p52 (**C**) nuclear accumulation in HFFs infected with RH (WT), Δ*myr1,* or Δ*myr1*::MYR1 complement parasites. Twenty-four hours post-infection, cells were fixed and labeled with DAPI (blue), anti-GAP45 (*T. gondii* [Tg]; red), and either anti-RelB or anti-p52 (green). Scale bars = 10 µm. Plots display the nuclear-to-cytoplasmic signal ratios of individual infected cells, with at least 300 cells analyzed per condition from at least three independent experiments. Infected cells are indicated by red arrows, and uninfected cells are indicated by white arrows. Black circles indicate the mean nuclear-to-cytoplasmic signal ratio of each independent biological replicate. Horizontal dashed lines indicate the overall mean. Statistical significance was determined by one-way ANOVA with Tukey’s multiple-comparison test performed on the means of independent biological replicates. ****P < 0.0001, ns = not significant.

MYR1-dependent export of dense granule effectors is a major driver of host-cell reprogramming, reshaping multiple core signaling pathways during infection (24). To identify dominant transcriptional regulators associated with this MYR1-dependent response, we performed *in silico* transcription-factor-enrichment analysis on host transcripts induced by WT parasites but not by the Δ*myr1* mutant. This analysis identified RelB as a top hit, implicating activation of the non-canonical NF-κB pathway (Fig. 1A). To validate this prediction, we measured the expression of the non-canonical NF-κB target genes BCL3 and BIRC3 by qRT-PCR. Both genes were significantly upregulated in cells infected with WT or Δ*myr1*::MYR1-complemented strains, whereas this response was lost in Δ*myr1*-infected cells, confirming MYR1-dependent downstream activation of the RelB/p52 pathway (Fig. S1).

Having confirmed transcriptional engagement of the non-canonical arm, we next examined RelB protein expression and localization directly. Immunofluorescence imaging of HFFs infected with type I (RH) or type II (ME49) *T. gondii* strains, combined with quantitative analysis, revealed that the RelB nuclear-to-cytoplasmic ratio increased to ~1.68 in type I-infected cells and, to a lesser extent, to ~1.26 in type II-infected cells (Fig. 1B; Fig. S2A). Both increases were significant relative to uninfected bystander cells within the same infected cultures (Fig. S4A and D). RelB nuclear accumulation was significantly reduced during infection with Δ*myr1* parasites, reaching a level statistically indistinguishable from that in the uninfected bystander cells, and was restored by MYR1 complementation (Δ*myr1::MYR1*). These findings indicate that MYR1-dependent effector export is required for RelB nuclear enrichment (Fig. 1B; Fig. S2A).

**Fig 2:**
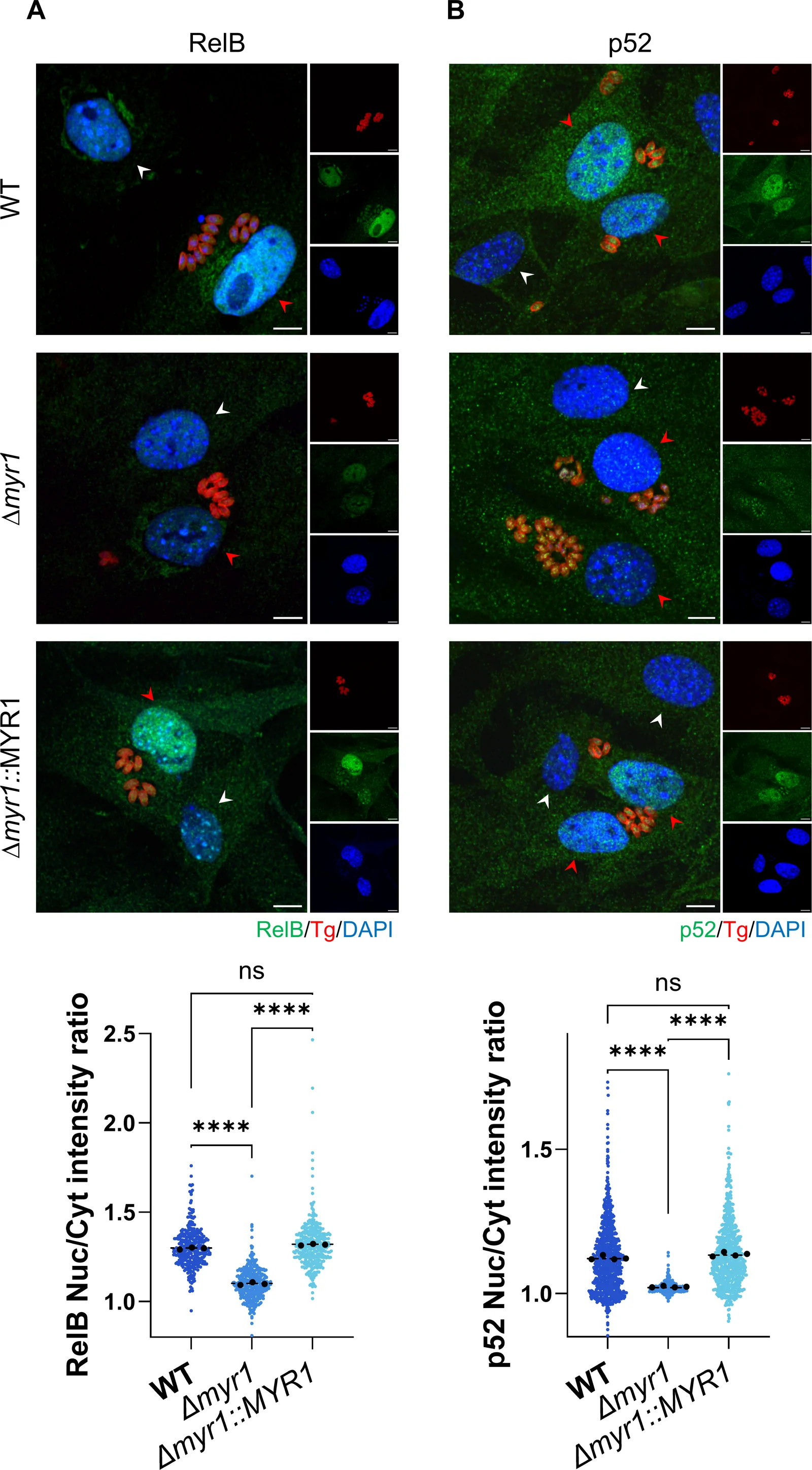
MYR1-dependent RelB and p52 nuclear accumulation in murine cells. (**A and B**) Representative images (top) and quantitative analysis (bottom) for RelB (**A**) and p52 (**B**) nuclear accumulation in MEFs infected with RH (WT), Δ*myr1,* or Δ*myr1*::MYR1 complement parasites. Twenty-four hours post-infection, cells were fixed and labeled with DAPI (blue), anti-GAP45 (*T. gondii* [Tg]; red), and either anti-RelB or anti-p52 (green). Scale bars = 10 µm. Plots display the nuclear-to-cytoplasmic signal ratios of individual infected cells, with at least 300 cells analyzed per condition from at least three independent experiments. Infected cells are indicated by red arrows, and uninfected cells are indicated by white arrows. Black circles indicate the mean nuclear-to-cytoplasmic signal ratio of each independent biological replicate. Horizontal dashed lines indicate the overall mean. Statistical significance was determined by one-way ANOVA with Tukey’s multiple-comparison test performed on the means of independent biological replicates. ****P < 0.0001, ns = not significant.

We next tested whether this phenotype is conserved across host species. Although uninfected mouse embryonic fibroblasts (MEFs) exhibited higher basal nuclear RelB than HFFs, infection with WT parasites of either type I or type II strains still significantly increased the nuclear-to-cytoplasmic RelB ratio to ~1.3 and ~1.2, respectively (Fig. 2A; Fig. S3A). This WT-induced increase was not observed during Δ*myr1* infection, where RelB ratios returned to the corresponding uninfected MEF baseline, and was restored upon MYR1 complementation (Δ*myr1::MYR1*), supporting a conserved MYR1-dependent mechanism (Fig. 2A; Fig. S3A and S4B and E).

Under basal conditions, RelB is sequestered in the cytoplasm by the NF-κB2 precursor p100, which functions as an IκB-like inhibitor through its C-terminal ankyrin repeat domains. Pathway activation triggers partial proteasomal processing of p100 into the active p52 subunit, facilitating the nuclear translocation of RelB/p52 heterodimers (37, 38). Consistent with this mechanism, infection with either type I or type II *T. gondii* strains increased nuclear p52 in both HFFs and MEFs (Fig. 1C and 2B; Fig. S2B and S3B). In all cases, nuclear p52 accumulation was significantly reduced during Δ*myr1* infection to levels statistically indistinguishable from those in the corresponding uninfected bystander cells and was restored by MYR1 complementation (Δ*myr1::MYR1*) (Fig. 1C and 2B; Fig. S2B, S3B, and S4A, B, D, and E). These findings indicate that MYR1-dependent effector export is required for the processing event associated with RelB nuclear entry.

To rule out a contribution from differential intracellular replication, we measured PV size 24 h post-infection. Vacuole sizes were comparable among WT, Δ*myr1*, and Δ*myr1::MYR1* parasites in both HFFs and MEFs and for both type I and type II backgrounds (Fig. S4C and F), indicating that the observed differences in nuclear RelB and p52 accumulation cannot be attributed to a growth phenotype.

### The known MYR1-dependent effector set cooperates to drive nuclear RelB accumulation

**Fig 3:**
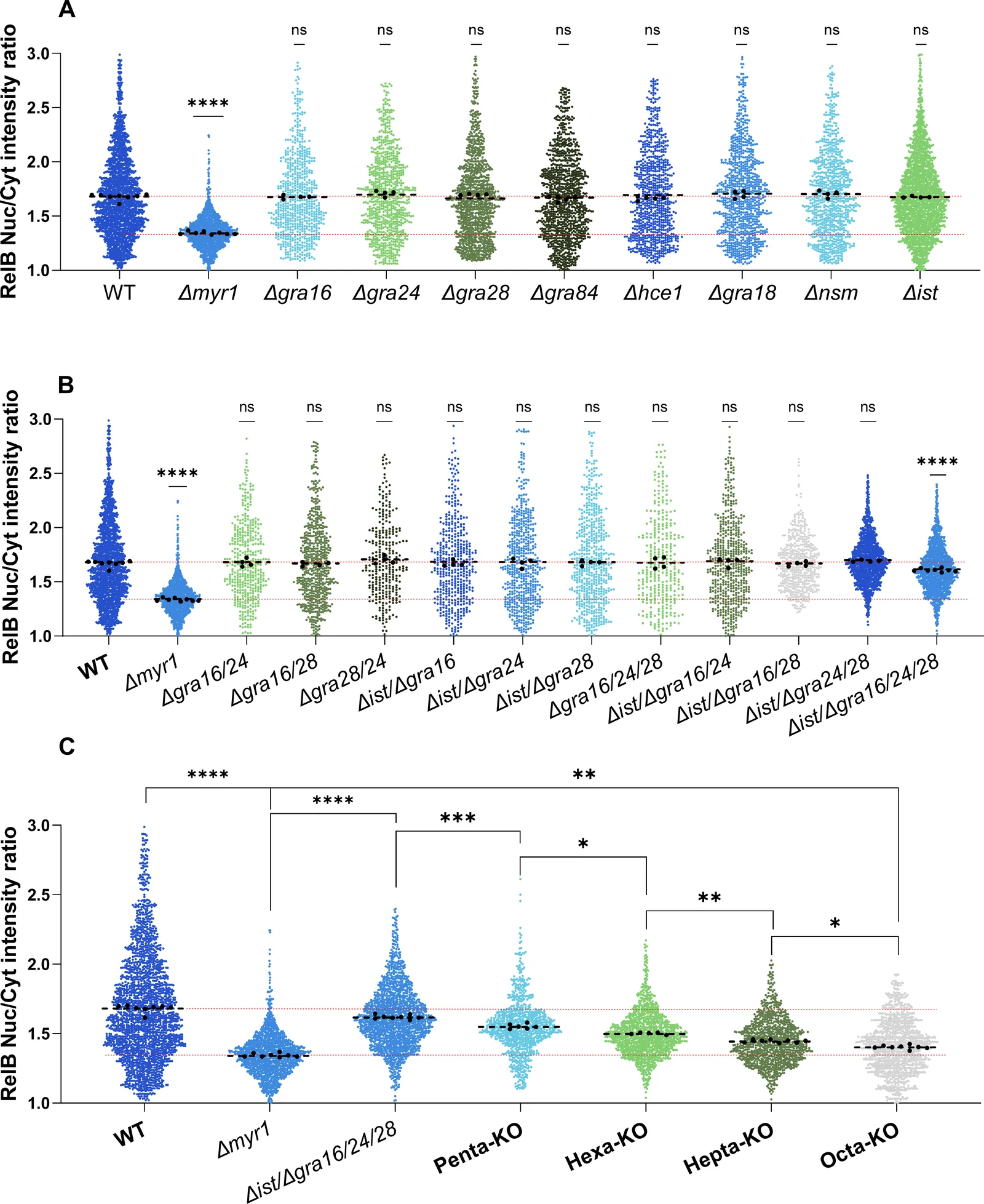
A suite of eight MYR1-dependent effectors cooperates to drive RelB nuclear accumulation. (**A**) Quantification of RelB nuclear accumulation in HFFs infected with single MYR1-dependent exported effector knockouts: Δ*gra16,* Δ*gra24,* Δ*gra28,* Δ*gra84,* Δ*ist,* Δ*hce1,* Δ*gra18,* and Δ*nsm*. (**B**) RelB nuclear-to-cytoplasmic intensity ratios in HFFs infected with various lower-order combinatorial knockout strains. (**C**) RelB nuclear-to-cytoplasmic intensity ratios in HFFs infected with higher-order combinatorial knockout parasites: quadruple knockout (Δ*ist/*Δ*gra16/*Δ*gra24/*Δ*gra28*), penta-KO (Δ*ist/*Δ*gra16/*Δ*gra24/*Δ*gra28/*Δ*gra84*), hexa-KO (Δ*ist/*Δ*gra16/*Δ*gra24/*Δ*gra28/*Δ*gra84/*Δ*hce1*), hepta-KO (Δ*ist/*Δ*gra16/*Δ*gra24/*Δ*gra28/*Δ*gra84/*Δ*hce1/*Δ*gra18*), and octa-KO (Δ*ist/*Δ*gra16/*Δ*gra24/*Δ*gra28/*Δ*gra84/*Δ*hce1/*Δ*gra18/*Δ*nsm*). (**A–C**) Plots display the nuclear-to-cytoplasmic intensity ratios of individual infected cells, with at least 300 cells analyzed per condition from at least three independent experiments. Black circles indicate the mean nuclear-to-cytoplasmic ratio of each independent biological replicate. Horizontal dashed lines indicate the overall mean. Statistical significance was determined by one-way ANOVA with Dunnett’s multiple-comparison test performed on the means of independent biological replicates. *P < 0.05, **P < 0.01, ***P < 0.001, ****P < 0.0001, ns = not significant.

To determine which MYR1-dependent effector(s) might drive this phenotype, we screened a panel of knockout parasites lacking individual secreted factors currently known to traffic to the host nucleus or cytoplasm. We tested single deletion mutants for GRA16, GRA18, GRA24, GRA28, GRA84, IST, NSM, and HCE1/TEEGR. Surprisingly, none of the single knockouts detectably reduced nuclear RelB accumulation, as all strains induced nuclear RelB at levels comparable to WT infection (Fig. 3A).

The lack of a phenotype in single knockouts suggested substantial functional redundancy, consistent with emerging evidence that *T. gondii* effectors often act in overlapping combinations to shape host transcriptional outputs (25, 26). To address this, we leveraged a panel of combinatorial knockout parasites that we previously generated while dissecting MYR1-dependent suppression of IFN-γ-responsive transcription, focusing on IST, GRA16, GRA24, and GRA28 (25). Percent reductions for combinatorial strains were calculated relative to the dynamic range between the WT ratio (~1.68) and the ∆*myr1* baseline (~1.34). While single mutants and lower-order combinations did not significantly reduce nuclear RelB accumulation, the quadruple knockout (Δ*ist/*Δ*gra16/*Δ*gra24/*Δ*gra28*) lowered the nuclear-to-cytoplasmic ratio to ~1.61, representing an approximately 20% shift toward the ∆*myr1* baseline (Fig. 3B). This partial reduction suggested that the phenotype is distributed beyond just this core set of IFN-γ-influencing effectors. We therefore expanded the set using our IFN-γ CRISPR screen as a guide and prioritized GRA84, which ranked 209th among negatively selected genes under IFN-γ pressure (25). Deleting GRA84 in the quadruple background (Δ*ist/*Δ*gra16/*Δ*gra24/*Δ*gra28/*Δ*gra84*) strengthened the defect, yielding a ratio of ~1.55, or an approximately 40% reduction in nuclear RelB relative to WT infection (Fig. 3C).

To further define the effector repertoire driving this phenotype, we expanded combinatorial mutagenesis to include additional MYR1-dependent effectors. We next deleted HCE1/TEEGR in the quintuple background, given its established role in modulating NF-κB-dependent transcription through EZH2 recruitment (15). The resulting hextuple mutant (Δ*ist/*Δ*gra16/*Δ*gra24/*Δ*gra28/*Δ*gra84/*Δ*hce1*) further reduced the ratio to ~1.49, an approximately 54% decrease relative to WT (Fig. 3C). We then targeted GRA18 (23). Although GRA18 is best known for stabilizing β-catenin, extensive crosstalk between Wnt and NF-κB signaling suggested that it could contribute to this phenotype (23, 39). Deleting GRA18 in the hextuple background produced a cumulative ratio of ~1.44, representing an approximately 70% reduction in nuclear RelB (Fig. 3C). Finally, to complete the analysis of the remaining candidate effector set, we deleted NSM in the septuple background. The resulting octuple knockout (Δ*ist/*Δ*gra16/*Δ*gra24/*Δ*gra28/*Δ*gra84/*Δ*hce1/*∆*gra18/*∆*nsm*) exhibited a ratio of ~1.4, an approximately 80% reduction compared to WT infection (Fig. 3C). To determine whether these reductions reflected impaired parasite growth rather than specific effects on non-canonical NF-κB signaling, we quantified vacuole size across the mutant panel. No significant differences were observed among strains (Fig. S5A). In addition, because the Δ*myr1* baseline remained above a ratio of 1.0, we quantified RelB nuclear-to-cytoplasmic ratios in uninfected bystander cells within the same infected cultures. RelB ratios in these bystander cells were consistent across infection conditions and were not statistically different from those measured in Δ*myr1*-infected cells (Fig. S5B). These data indicate that the Δ*myr1* phenotype approximates the basal, non-activated state rather than reflecting residual infection-induced RelB activation. Thus, the progressive reduction in RelB accumulation observed across the combinatorial mutants cannot be explained by differences in parasite growth or basal RelB localization. Collectively, these data confirm substantial functional redundancy and indicate that this suite of eight MYR1-dependent effectors cooperates to drive most of the non-canonical NF-κB response during *T. gondii* infection.

### MYR1-dependent TRAF3 depletion stabilizes NIK and drives p100 processing

**Fig 4:**
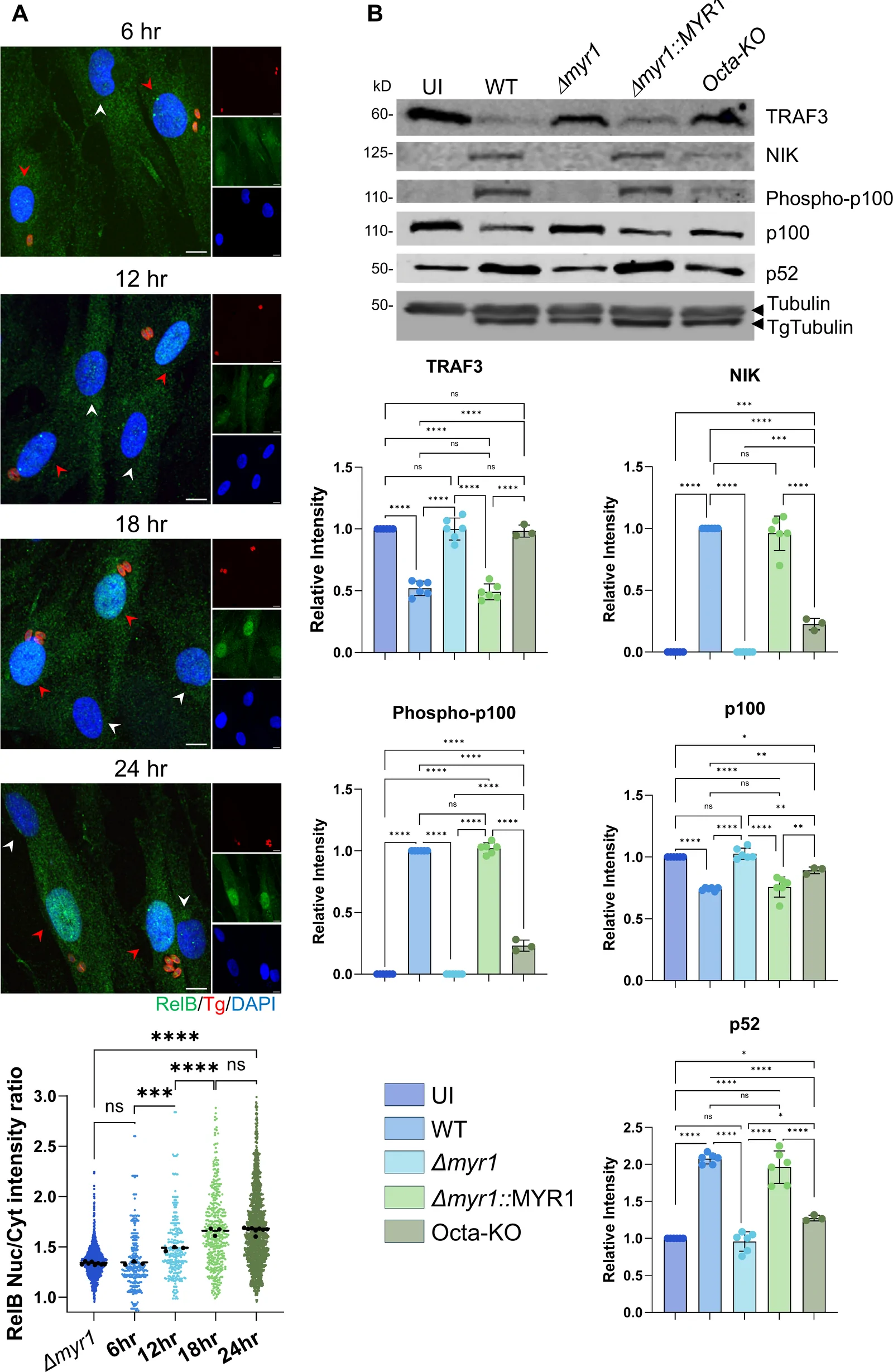
*T. gondii* activates the non-canonical NF-κB pathway through MYR1-dependent TRAF3 depletion and NIK stabilization. (**A**) Representative images (top) and quantitative analysis (bottom) for RelB nuclear accumulation in HFFs infected with RH (WT) parasites over a 24-h time course. At 6, 12, 18, and 24 h post-infection, cells were fixed and labeled with DAPI (blue), anti-GAP45 (*T. gondii* [*Tg*]; red), and anti-RelB (green). RelB nuclear-to-cytoplasmic ratios in Δ*myr1*-infected cells at 24 h post-infection are shown as a reference baseline. Scale bars = 10 µm. Infected cells are indicated by red arrows, and uninfected cells are indicated by white arrows. Plots display the nuclear-to-cytoplasmic intensity ratios of individual infected cells, with at least 300 cells analyzed per condition from at least three independent experiments. Black circles indicate the mean nuclear-to-cytoplasmic ratio of each independent biological replicate. Horizontal dashed lines indicate the overall mean. Statistical significance was determined by one-way ANOVA with Tukey’s multiple-comparison test performed on the means of independent biological replicates. ***P < 0.001, ****P < 0.0001, ns = not significant. (**B**) Immunoblot analysis (top) and corresponding quantification (bottom) of TRAF3, NIK, p100 phosphorylation, and p100-to-p52 processing in HFFs that were uninfected (UI) or infected with RH (WT), Δ*myr1,* Δ*myr1*::MYR1 complement parasites, or octa-KO (Δ*ist/*Δ*gra16/*Δ*gra24/*Δ*gra28/*Δ*gra84/*Δ*hce1/*Δ*gra18/*Δ*nsm*). Lysates collected 24 h post-infection were resolved by SDS-PAGE and probed with specific primary antibodies; β-tubulin served as a loading control. Band intensities were measured using ImageJ and normalized to β-tubulin. Data are presented as mean ± SD from at least three biological replicates. Statistical significance for all panels was determined using a one-way ANOVA with Tukey’s multiple-comparison test; *P < 0.05, **P < 0.01, ***P < 0.001, ****P < 0.0001, ns = not significant.

The non-canonical NF-κB pathway depends on NIK stabilization and proteasome-dependent processing of p100, rather than rapid IκB degradation, and therefore typically displays slower, more sustained activation kinetics than the canonical pathway (33, 40). To test whether *T. gondii* infection follows similar dynamics, we quantified nuclear RelB levels in RH-infected HFF cells every 6 h over a 24 h period. RH was used for this analysis given its more robust induction of nuclear RelB relative to ME49 (Fig. 1B; Fig. S2A). This analysis revealed a gradual, progressive increase in nuclear RelB that reached its highest measured level at 18 h post-infection and remained elevated through 24 h, consistent with the characteristic kinetics of the non-canonical NF-κB arm (Fig. 4A) (41).

To elucidate the mechanism by which *T. gondii* drives RelB/p52 activation, we examined upstream components of the non-canonical NF-κB pathway. In this pathway, TRAF3 constrains NIK abundance by promoting its constitutive degradation, and loss of TRAF3 stabilizes NIK to initiate downstream signaling (35, 42). Consistent with this mechanism, lysates from cells infected with WT or ∆*myr1*::MYR1 complement parasites showed a marked reduction in TRAF3 protein levels compared to uninfected or ∆*myr1*-infected cells (Fig. 4B). This MYR1-dependent depletion of TRAF3 coincided with robust NIK stabilization, p100 phosphorylation, and increased p52 processing (Fig. 4B). To determine whether the eight-effector suite identified above also accounts for these upstream biochemical events, we extended the analysis to the octuple knockout strain (Δ*ist*/Δ*gra16*/Δ*gra24*/Δ*gra28*/Δ*gra84*/Δ*hce1*/Δ*gra18*/Δ*nsm*). Despite retaining a functional MYR1 translocon, octa-KO-infected cells failed to deplete TRAF3, with levels indistinguishable from uninfected or Δ*myr1*-infected controls, and showed a near-complete loss of NIK stabilization and p100 phosphorylation together with substantially diminished p100-to-p52 processing (Fig. 4B). The small residual p52 signal in octa-KO-infected cells (~30% above the uninfected baseline) closely parallels the residual nuclear RelB accumulation observed in this same strain (Fig. 3C). Together, these results indicate that *T. gondii* engages the non-canonical NF-κB pathway, with MYR1-dependent effector export required for TRAF3 loss, NIK stabilization, and p100-to-p52 processing, and that the same eight-effector suite that drives nuclear RelB accumulation is also responsible for the bulk of these upstream biochemical events (Fig. 4B).

## DISCUSSION

The co-evolutionary struggle between *T. gondii* and the host immune system is perhaps most clearly defined by the parasite’s sophisticated manipulation of the NF-κB signaling axis. While much of our understanding has centered on the subversion of canonical p65/p50 signaling, our findings identify activation of the non-canonical NF-κB pathway as a previously underappreciated outcome of the tachyzoite stage of infection. We show that *T. gondii* induces nuclear accumulation of RelB and p52 in human and mouse fibroblasts across both type I and type II genetic backgrounds, although RelB accumulation is lower following type II infection than type I infection. Importantly, infection-induced RelB and p52 nuclear accumulation are dependent on MYR1-mediated effector export, as Δ*myr1* infection returned RelB and p52 levels to those observed in uninfected bystander cells and MYR1 complementation restored the response. Thus, dense granule translocation is a major determinant of the non-canonical response.

Our analysis of upstream pathway structure suggests that *T. gondii* engages non-canonical NF-κB at the level of the NIK regulatory checkpoint. WT infection triggers depletion of TRAF3, stabilization of NIK, and subsequent phosphorylation and processing of p100, all of which are abolished in the absence of MYR1. This model is strongly supported by the established architecture of non-canonical NF-κB signaling, in which TRAF3 serves as a key negative regulator of NIK abundance and p100 processing to p52, thereby licensing RelB/p52 nuclear signaling (35, 43). Mechanistically, the MYR1-dependent effector suite implicated here is composed of established transcriptional modulators that reprogram host gene expression, raising the possibility that *T. gondii* engages non-canonical NF-κB signaling through indirect remodeling of host gene expression rather than through a single direct interaction with pathway components (44). One potential model is that coordinated transcriptional changes alter the abundance of upstream regulators such as TRAF3, TRAF2, cIAP1/2, or other checkpoint proteins, thereby lowering the threshold for NIK accumulation (33, 35). However, the precise molecular mechanisms linking MYR1-dependent effectors to TRAF3 depletion and NIK stabilization remain to be determined.

The observation that non-canonical RelB/p52 activation is conserved across both type I and type II strains, while canonical RelA/p65 activation is a unique feature of type II infection, suggests a tiered strategy for host NF-κB reprogramming (28, 29). In this model, the engagement of the non-canonical pathway could represent a baseline component of host cell subversion, driven by a distributed network of eight MYR1-dependent effectors common to both lineages. In contrast, the GRA15-mediated activation of RelA/p65 in type II strains overlays a specialized inflammatory signature onto this basal state (28). One potential consequence of this dual activation is the formation of RelA-RelB heterodimers, which have been reported to possess distinct DNA-binding properties and to participate in regulatory feedback that can temper acute inflammatory responses (45). By maintaining a conserved non-canonical response across lineages while varying the canonical input, the parasite may differentially shape the host signaling equilibrium, potentially balancing host cell viability with the evasion of sterile immunity across diverse genetic backgrounds.

A central conceptual finding is that non-canonical NF-κB activation is not attributable to a single exported effector. Across a panel of parasites lacking individual MYR1-dependent effectors, none of the single deletions significantly diminished nuclear RelB accumulation. Instead, the phenotype emerged through a cumulative, multi-effector regulatory network, with progressively larger defects observed as additional effectors were deleted in higher-order mutant backgrounds. This progressive reduction suggests that the eight effectors identified here, GRA16, GRA18, GRA24, GRA28, GRA84, IST, NSM, and HCE1/TEEGR, do not merely provide redundancy in the classical sense but rather function through additive cooperation to reach a signaling threshold. This distributed logic mirrors the strategies described for the suppression of IFN-γ-responsive transcription and the tuning of canonical NF-κB outputs, extending this principle to pathway choice within the NF-κB system itself (15, 25, 26).

Understanding why *T. gondii* invests so heavily in multi-node targeting benefits from considering the host’s own architecture. Innate signaling and transcriptional circuits are characterized by profound transcriptional robustness, shaped by evolution to maintain stable outputs despite genetic variation and environmental noise (46, 47). Population-scale human genome analyses support this robustness, identifying predicted biallelic loss-of-function genotypes in a subset of NF-κB pathway genes, including core components such as p65 and IKKα, as well as regulators linked to non-canonical pathway control, such as ZFP91 and TRIM55 (48–51). Although some of these genotypes may have incompletely characterized clinical consequences or present as specific immune phenotypes rather than being uniformly benign, their presence in ostensibly healthy populations underscores the inherent plasticity of the host signaling network. This stability is maintained through multiple layers of control, including functional redundancy between transcription factor paralogs and the existence of parallel signaling cascades (47). Consistent with this idea, NF-κB upstream signaling modules include seemingly redundant components in which loss of one family member can be partially compensated for by others, as illustrated by partial compensation for IKKβ loss by IKKα *in vivo* (52). In such a stable system, a parasite strategy that relies on a single effector to trigger a specific molecular change would likely be buffered and neutralized by the host’s internal backups (33, 53). By deploying a suite of effectors with overlapping activities, *T. gondii* can push multiple control points simultaneously to successfully and appropriately modulate this host function, converting a stable circuit into one that is permissive for sustained non-canonical signaling.

The biological consequences of parasite-induced non-canonical NF-κB activation remain to be determined. Prior studies established that RelB is required for protective immunity to *T. gondii in vivo* (36), underscoring the importance of this pathway during infection. However, how parasite-driven RelB/p52 activation in fibroblasts contributes to infection outcomes remains unclear. *In vitro*, phenotypes associated with MYR1-dependent effector loss can be highly context-dependent and may become apparent only under defined host stimulation conditions. For example, we previously showed that Δ*myr1* and higher-order effector mutants exhibit host-cell death and premature parasite egress following IFN-γ stimulation, whereas these phenotypes are minimal under basal conditions (25). The relevant physiological contexts in which parasite-induced RelB/p52 activation affects host-cell or parasite outcomes remain to be defined. Future studies examining RelB/p52-dependent transcriptional and functional outputs across immune and non-immune cell types will be important for understanding how this pathway shapes host-parasite interactions.

Several important questions follow from the results reported here. First, defining the RelB-dependent transcriptional program specifically induced during *T. gondii* infection will be essential to determine whether parasite-driven RelB activation reinforces, diverts, or partially rewires canonical immune outputs. Second, although the eight-effector knockout substantially reduced nuclear RelB accumulation, it did not fully phenocopy Δ*myr1* infection, raising the possibility that additional MYR1-dependent exported factors or indirect host-mediated inputs contribute to the remaining MYR1-dependent activity. Importantly, because Δ*myr1*-infected cells were statistically indistinguishable from uninfected bystander cells, this residual activity in the octuple knockout does not indicate a separate MYR1-independent response to parasite material but rather an incomplete accounting of the MYR1-dependent effector repertoire. Future experiments that pair RelB loss of function with transcriptomic profiling and phenotypic readouts will be essential to clarify how this axis shapes the balance between parasite fitness and host cell fate.

## MATERIALS AND METHODS

### Parasite and host cell culture

*T. gondii* tachyzoites were serially passaged in human foreskin fibroblast (HFF) monolayers cultured in D10 medium (Dulbecco’s modified Eagle’s medium [DMEM; Invitrogen]) supplemented with 10% HyClone Cosmic calf serum (Cytiva), 10 µg/mL gentamicin (Thermo Fisher Scientific), and 2 mM glutamine (Thermo Fisher Scientific). MEF cells were maintained in D10. All strains and host cell lines were determined to be mycoplasma-negative using the e-Myco Plus Kit (Intron Biotechnology). Strains used in this study are listed in Table S1. Gene disruptant lines were generated using CRISPR/Cas9 (54), as described below. Parasite lines generated in other studies include RHΔ*hxgprt*Δ*ku80* (55), ME49 *FLuc* (56), and RH Δ*gra84* (22).

### Plasmid construction and genome editing

Plasmids were generated by site-directed mutagenesis of existing plasmids or assembled from DNA fragments by the Gibson method (57). All plasmids used in this study are listed in Table S2.

### Primers

All primers were synthesized by Sigma. All CRISPR/Cas9 plasmids used in this study were derived from the single-guide RNA (sgRNA) plasmid pSAG1:CAS9-GFP, U6:sgUPRT (54) by Q5 site-directed mutagenesis (New England Biolabs) to alter the 20-nt sgRNA sequence, as described previously (58). Primers for plasmids are listed in Table S3.

### Parasite transfection

Following natural egress, freshly harvested extracellular parasites were transfected with plasmids as previously described (54). Briefly, approximately 2 × 10^7^ parasites were resuspended in 370 µL cytomix buffer and mixed with approximately 30 µL purified plasmid or amplicon DNA in a 4-mm gap BTX cuvette. Parasites were electroporated using a BTX ECM 830 electroporator (Harvard Apparatus) with the following settings: 1,700 V, 176 µs pulse length, two pulses, and a 100 ms interval between pulses. Transgenic parasites were selected by outgrowth under drug selection as needed using mycophenolic acid (25 µg/mL) plus xanthine (50 µg/mL) (MPA/Xa), pyrimethamine (3 µM), chloramphenicol (40 µM), 5-fluorodeoxyuridine (10 µM) (Sigma), or phleomycin (5 µg/mL) (InvivoGen). Stable clones were isolated by limiting dilution on HFF monolayers in 96-well plates.

### Generation of quintuple, hextuple, septuple, and octuple knockout strains

Because available selectable markers were limiting, 5- through 8-gene knockout strains were generated by fluorescence-activated cell sorting and subcloning without drug selection. All sgRNA plasmids used in this study are based on the pSAG1:CAS9-GFP, U6:sgUPRT backbone (54), which constitutively expresses a Cas9-GFP fusion. The same Cas9-GFP signal was used as the sorting marker in every round of multi-gene knockout generation.

For each new gene deletion, extracellular parasites of the parental knockout strain were co-transfected with the relevant sgRNA-expressing Cas9-GFP plasmid and an amplicon donor DNA at a 1:3 (plasmid:donor) molar ratio. Donor amplicons were generated by PCR from genomic DNA of the corresponding single knockout strain, amplifying the integrated selectable cassette flanked by ~500 bp of upstream and downstream homology (Fig. S6A). Transfected parasites were recovered on HFF monolayers, mechanically released, and GFP+ live single parasites were sorted directly into 96-well HFF monolayers for subcloning. Clones were verified by diagnostic PCR across the targeted locus (Fig. S6B through F).

### Whole genome sequencing and CRISPR off-target analysis

Genomic DNA from the *T. gondii* RH octa-knockout strain was submitted to Admera Health (South Plainfield, NJ) for library preparation and sequencing. Libraries were prepared using the Roche KAPA HyperPrep Kit and sequenced on an Illumina NovaSeq X Plus platform using paired-end sequencing (151 bp reads).

Raw sequencing reads were quality-assessed using FastQC and trimmed using fastp (v1.3.3). Trimmed reads were aligned to the *T. gondii* GT1 reference genome (ToxoDB release 68) using BWA-MEM (v0.7.19). Alignment files were processed using SAMtools to generate sorted, indexed BAM files.

Successful gene deletion was confirmed by searching all 75.7 million sequencing reads for internal sequences specific to each of the eight target genes (GRA84, GRA16, GRA24, IST, NSM, GRA18, TEEGR/HCE1, and GRA28). Zero reads containing any target gene sequence were detected, confirming complete deletion of all eight loci (Fig. S7).

For off-target analysis, potential Cas9 cleavage sites were predicted for each guide RNA by searching all annotated coding sequences (exons) in the GT1 reference genome for sequences with up to five mismatches relative to each guide. Sites were scored based on mismatch position, with mismatches in the seed region (positions 9–20, proximal to the PAM) weighted more heavily. The top five predicted off-target sites per guide RNA were selected for further analysis. Variants were called at all predicted off-target sites using bcftools and filtered for high-confidence calls (quality score ≥100). No insertions or deletions were detected at any of the 40 predicted off-target sites, indicating that CRISPR-Cas9 editing did not introduce detectable off-target mutations in coding regions of the genome (Table S4).

### Immunofluorescence assay (IFA) and image analysis

Host cells were seeded onto glass coverslips in 24-well plates and cultured for 24 h prior to infection. Cells were infected the following day with *T. gondii* (1 × 10^5^ parasites per well). At 24 h post-infection, infected cells were fixed with 4% formaldehyde for 10 min at room temperature, washed with phosphate-buffered saline (PBS), permeabilized with 0.2% Triton X-100 for 10 min, and blocked in PBS containing 10% normal goat serum (Thermo) (PBS with 10% NGS).

Coverslips were washed four times in PBS (5 min per wash) and incubated with primary antibodies diluted in blocking buffer overnight at 4°C. The following primary antibodies were used: rabbit anti-RelB (1:300; Proteintech, 25027-1-AP), mouse anti-NF-κB p52 (1:100; Santa Cruz Biotechnology, sc-7386), and mouse anti-GAP45 or rabbit anti-GAP45 (kindly provided by Dr. D. Etheridge, University of Georgia, USA). Samples were washed four times in PBS (5 min per wash) and incubated with secondary antibodies diluted in blocking buffer containing Hoechst 33342 (1 µg/mL). Secondary antibodies were goat anti-rabbit Alexa Fluor 488 (1:2,000) and goat anti-mouse Alexa Fluor 488 (1:2,000) (Invitrogen). *T. gondii*-containing vacuoles were stained with anti-GAP45 and visualized with Alexa Fluor 568 (Invitrogen). Following secondary staining, coverslips were washed four times in PBS (5 min per wash) and mounted on slides using ProLong Gold Antifade Reagent (Invitrogen).

Samples were imaged using a Plan-Apochromat 20× objective (Zeiss) on a 3i Marianas Plus CSU-W1 SoRa super-resolution spinning-disk confocal microscope equipped with a LaserStack (3i) laser source and a Prime 95B CMOS digital camera (Teledyne Photometrics). Images were acquired using SlideBook software.

Raw immunofluorescence images were exported from SlideBook as single-channel 16-bit TIFF files and analyzed in CellProfiler (version 4.2.8). For each field of view, matched 3-channel image sets (DAPI, GAP45, and RelB or p52) were processed together. Host nuclei were segmented from the DAPI channel using size-based filtering to exclude debris and merged nuclei. Parasites were segmented independently from the GAP45 channel using size-based gating. Whole-cell regions were approximated by expanding outward from each nucleus using the RelB or p52 signal, which is distributed throughout the host cell. Cytoplasmic regions were then defined by subtracting the nuclear mask from the whole-cell mask. Parasites were assigned to host cells to classify cells as infected or uninfected, and this classification was propagated to the corresponding nuclei. Parasite regions were excluded from cytoplasmic measurements to avoid contamination of host fluorescence measurements by parasite-derived signal.

RelB or p52 fluorescence was quantified as mean pixel intensity within the nuclear and cytoplasmic compartments, and a nuclear-to-cytoplasmic mean intensity ratio was calculated for each cell. Nuclear-to-cytoplasmic ratios were quantified in at least 100 infected cells per group in each biological replicate across at least three independent experiments.

To quantify vacuole size, the same raw images were reanalyzed in CellProfiler using the GAP45 channel. Parasites were segmented using global minimum cross-entropy thresholding with size-based filtering, and objects touching the image border were excluded. Parasite objects within 3 pixels of one another were merged to define individual vacuoles. Vacuole area was measured using CellProfiler object size and shape measurements and converted from pixels to µm² using the image pixel-to-micron conversion factor.

Measurement outputs were exported as spreadsheets, organized in Microsoft Excel, provided as Table S5, and analyzed and plotted in GraphPad Prism.

### Immunoblotting

HFFs grown in T-12.5 flasks were infected with 5 × 10^6^ parasites per flask. At 24 h post-infection, monolayers were washed with PBS, scraped into ice-cold PBS, and pelleted at 500 × *g*. Cell pellets were lysed in RIPA buffer (50 mM Tris-HCl, pH 7.4; 150 mM NaCl; 1% Triton X-100; 0.5% sodium deoxycholate; 0.1% SDS) supplemented with 1 × Halt protease and phosphatase inhibitor cocktail.

Protein samples were prepared in Laemmli buffer containing 100 mM dithiothreitol, boiled for 5 min, resolved by SDS-PAGE on 10% polyacrylamide gels, and transferred to nitrocellulose membranes. Membranes were blocked in PBS containing 2.5% fat-free milk, then incubated with primary antibodies diluted in blocking buffer containing 0.1% Tween 20. Membranes were washed in PBS with 0.1% Tween 20 and incubated with goat IRDye-conjugated secondary antibodies (LI-COR Biosciences) diluted in blocking buffer, as indicated in the figure legends. After additional washes, membranes were scanned on a LI-COR Odyssey imaging system (LI-COR Biosciences). Band intensities were quantified using ImageJ.

The primary antibodies used were rabbit anti-phospho-NF-κB2 p100 (Ser866/870) (1:1,000; Cell Signaling Technology, #4810), rabbit anti-NF-κB2 p100/p52 (1:1,000; Cell Signaling Technology, #4882), rabbit anti-NIK (1:1,000; Cell Signaling Technology, #4994), rabbit anti-TRAF3 (1:1,000; Cell Signaling Technology, #4729), and mouse anti-β-tubulin (Developmental Studies Hybridoma Bank, AB_528499), which detects both parasite and host β-tubulin and was used as a loading control.

### Transcription factor enrichment analysis

To identify candidate transcription factors associated with MYR1-dependent host transcriptional reprogramming, we used the previously published transcriptomic data set of Naor et al. (24), which compared host gene expression in HFFs infected with WT or Δ*myr1* parasites to uninfected controls. We defined MYR1-dependent genes as those with a log2 fold change ≥2 in WT-infected cells but not in Δ*myr1*-infected cells, yielding a final list of 644 genes.

This gene list was submitted to Enrichr (https://maayanlab.cloud/Enrichr/) (59–61) and analyzed against the ChEA transcription factor target library, which curates transcription factor-target relationships from published ChIP-seq and related data sets. Enrichment was assessed by Fisher’s exact test, and transcription factors were ranked by Enrichr’s combined score. The top five transcription factors by combined score are shown in Fig. 1A, with bar length proportional to the combined score.

### Real-time PCR

Cells were left uninfected or infected with parasites for 24 h. Samples were lysed, and RNA was extracted using a ZYMO Quick-RNA Miniprep Kit per the manufacturer’s instructions. Complementary DNA (cDNA) was prepared using a Bio-Rad iScript cDNA Synthesis Kit (Bio-Rad Laboratories, Inc.) per the manufacturer’s instructions. Real-time PCR was performed using TB Green Advantage qPCR premix (Takara Bio USA, Inc.) per the manufacturer’s instructions. Data acquisition was performed using the CFX96 Real-Time System (Bio-Rad) and analyzed using CFX Maestro Software (Bio-Rad). Primers are listed in Table S3. Comparative cycle threshold values were used to evaluate fold changes in transcripts using the β-actin gene as an internal transcript control.

### Statistical analysis

Statistical analysis for each experiment was performed on biological replicate means in PRISM (GraphPad). Replicates, significance values, and tests performed are included in the figure legends.

## Data Availability

Sequencing reads were deposited in the Sequence Read Archive Repository at the National Center for Biotechnology Information, SRA accession no. PRJNA1475477.

## SUPPLEMENTAL MATERIAL

Supplemental figures mbio.01652-26-s0001.pdf Fig. S1 to S7.

Table S1 mbio.01652-26-s0002.docx *T. gondii* strains used in this study.

Table S2 mbio.01652-26-s0003.docx Plasmids used in this study.

Table S3 mbio.01652-26-s0004.xlsx Primers used in this study.

Table S4 mbio.01652-26-s0005.xlsx Predicted Cas9 off-target sites and variant calling results for the octa-knockout strain sgRNAs.

Table S5 mbio.01652-26-s0006.xlsx Source data.

ASM does not own the copyrights to Supplemental Material that may be linked to, or accessed through, an article. The authors have granted ASM a non-exclusive, world-wide license to publish the Supplemental Material files. Please contact the corresponding author directly for reuse.
