# Supplementary material for: A suite of eight Toxoplasma gondii effectors cooperates to activate the non-canonical NF-κB pathway
Source: mBio. 2026 Aug 28;17(10):e01652-26. doi: 10.1128/mbio.01652-26 (PMC13712946; doi:10.1128/mbio.01652-26)
Supplement: Supplementary file 1 [file mbio.01652-26-s0001.pdf]

Figure. S1

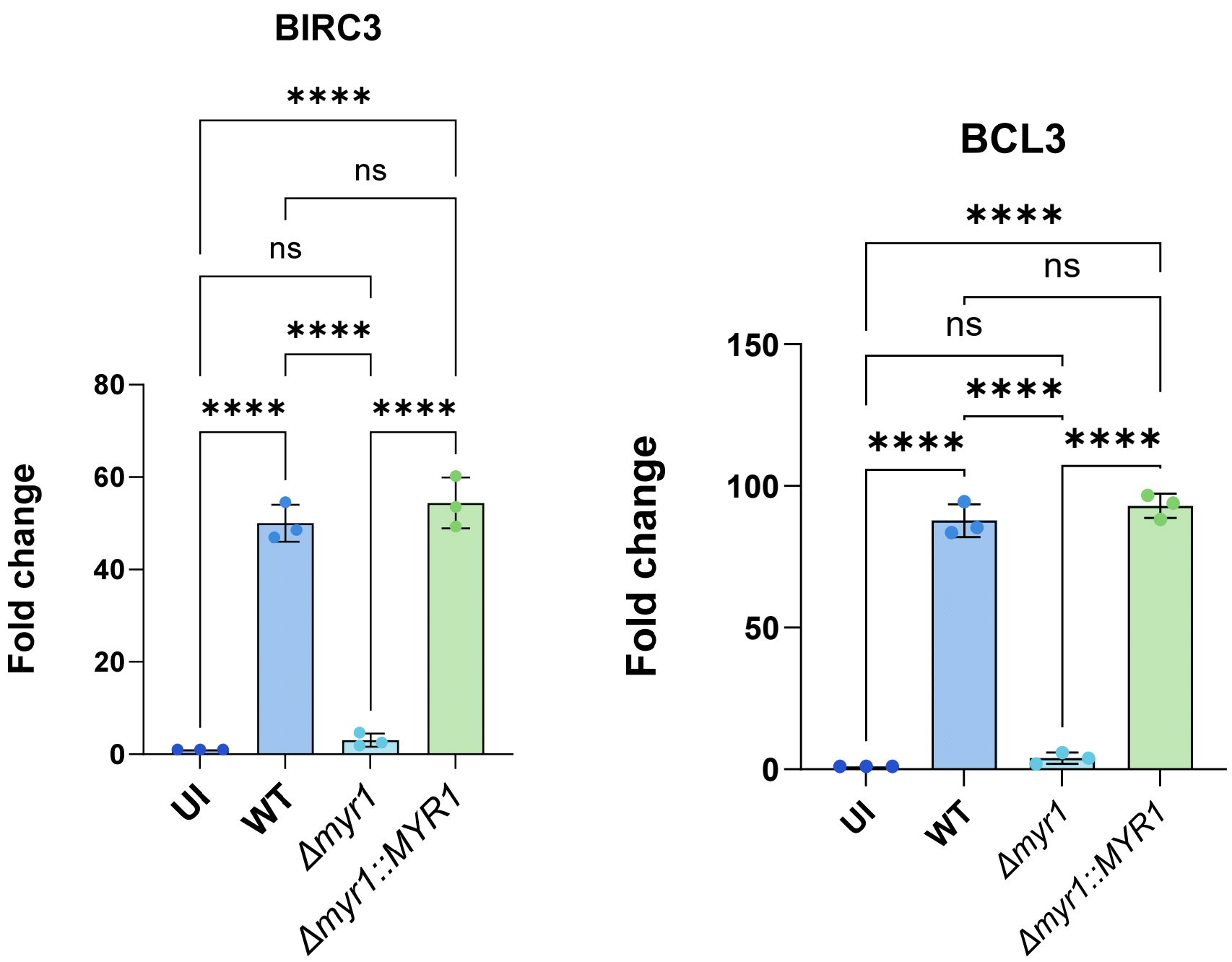
BIRC3
Fold change
0
20
40
60
80
****
ns
ns
****
****
****
UI
WT
Δmyr1
Δmyr1::MYR1
BCL3
Fold change
0
50
100
150
****
ns
ns
****
****
****
UI
WT
Δmyr1
Δmyr1::MYR1

Figure S1. qRT-PCR analysis of non-canonical NF-κB induced transcripts BCL3 and BIRC3.

RNA was isolated from HFF cells infected with RH (WT), ∆*myr1*, or ∆*myr1::MYR1* complemented parasites for 24 h and analyzed by qRT-PCR. Comparative cycle threshold (Ct) values were used to evaluate fold change in transcripts using β-actin (ActB) as an internal control. Data are plotted as fold change ± SEM from at least three independent experiments per gene. Statistical significance was determined using one-way ANOVA with Tukey's multiple comparison test. ****P < 0.0001.

Figure. S2

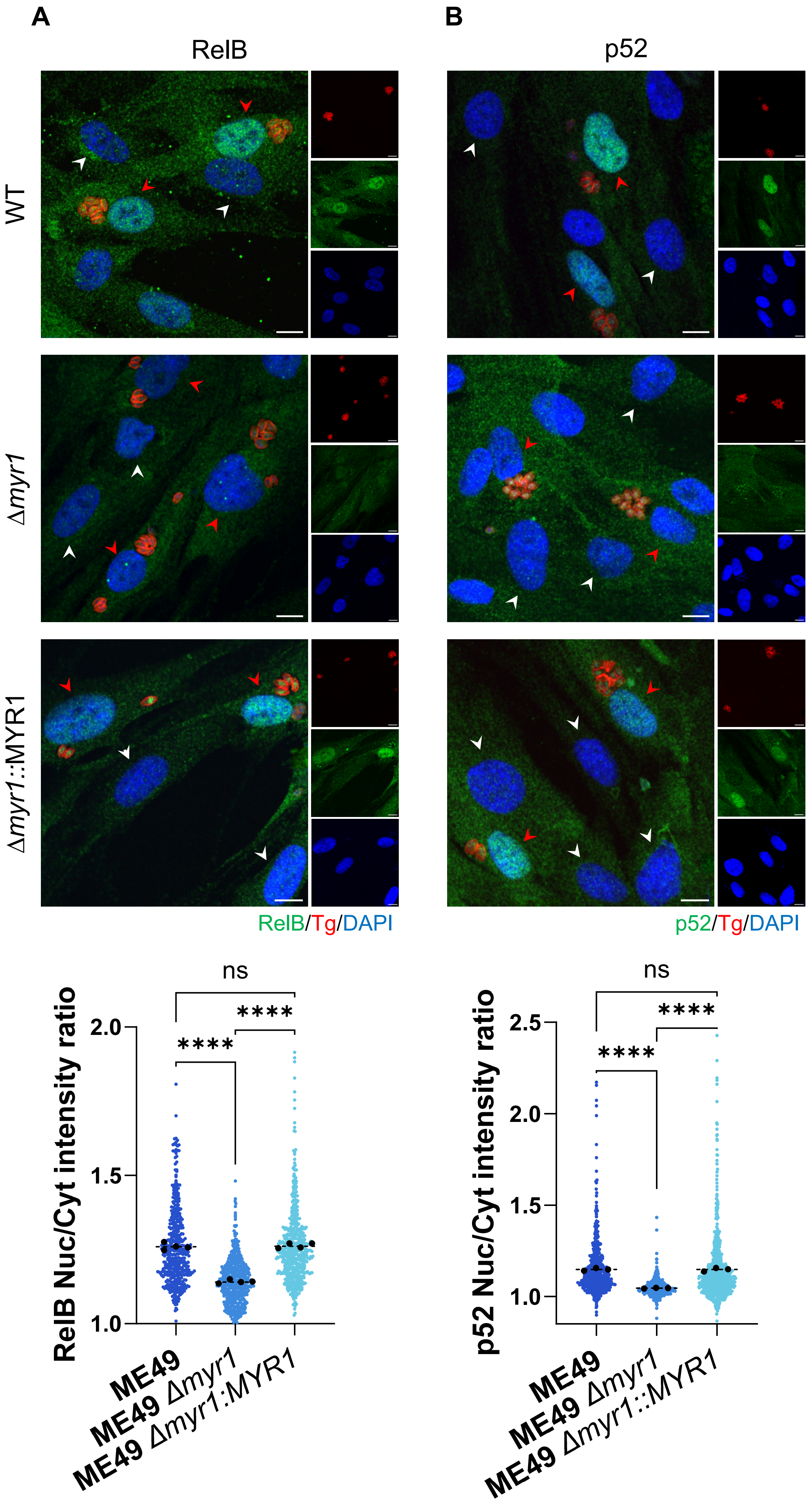

A
RelB
WT
Δmyr1
Δmyr1::MYR1
RelB/Tg/DAPI
B
p52
p52/Tg/DAPI
ns
****
****
RelB Nuc/Cyt intensity ratio
2.0
1.5
1.0
ME49
ME49 Δmyr1
ME49 Δmyr1:MYR1
ns
****
****
p52 Nuc/Cyt intensity ratio
2.5
2.0
1.5
1.0
ME49
ME49 Δmyr1
ME49 Δmyr1::MYR1

Figure S2. Conservation of MYR1-dependent non-canonical NF-κB activation in Type II (ME49) parasites.

A-B) Representative images (top) and quantitative analysis (bottom) for RelB **(A)** and p52 **(B)** nuclear accumulation in HFFs infected with Type II ME49 (WT), Δ*myr1* or Δ*myr1*::MYR1 complement parasites. Twenty-four hours post-infection, cells were fixed and labeled with DAPI (blue), anti-GAP45 (red), and either anti-RelB or anti-p52 (green). Scale bars = 10 μm. Plots display the nuclear-to-cytoplasmic intensity ratios of individual infected cells, with at least 300 cells analyzed per condition from at least three independent experiments. Black circles indicate the mean nuclear-to-cytoplasmic ratio of each independent biological replicate. Horizontal dashed lines indicate the overall mean. Statistical significance was determined using a one-way ANOVA with Tukey's multiple comparison test; ****$P < 0.0001$, ns = not significant.

Figure. S3

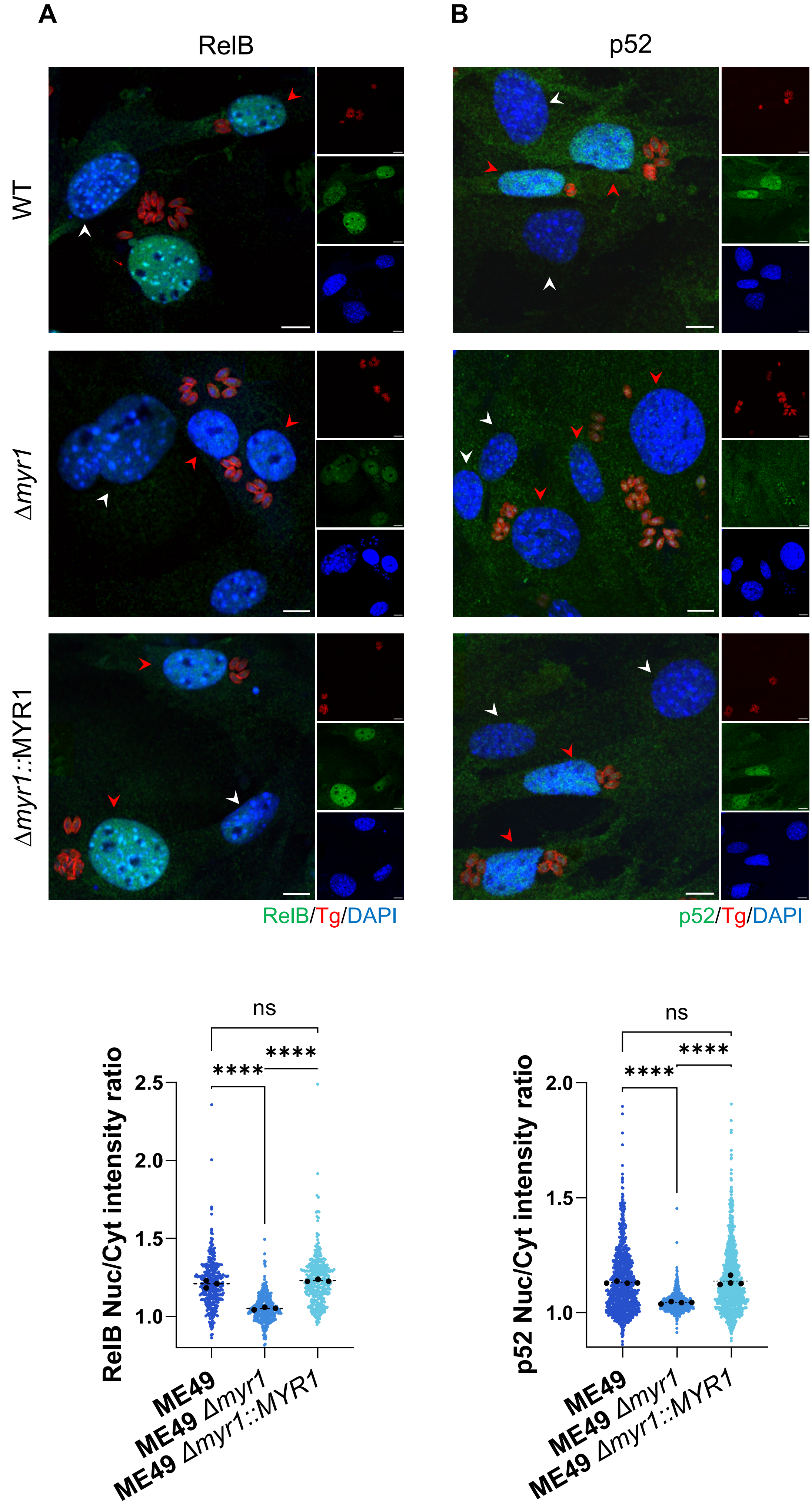

A
B
RelB
p52
WT
Δmyr1
Δmyr1::MYR1
RelB/Tg/DAPI
p52/Tg/DAPI
ns
****
****
RelB Nuc/Cyt intensity ratio
2.5
2.0
1.5
1.0
p52 Nuc/Cyt intensity ratio
2.0
1.5
1.0
ME49
ME49 Δmyr1
ME49 Δmyr1::MYR1

Figure S3. Type II (ME49) *T. gondii* induces MYR1-dependent RelB and p52 accumulation in murine cells.

A-B) Representative images (top) and quantitative analysis (bottom) for RelB **(A)** and p52 **(B)** nuclear accumulation in MEFs infected with Type II ME49 (WT), Δ*myr1* or Δ*myr1*::MYR1 complement parasites. Twenty-four hours post-infection, cells were fixed and labeled with DAPI (blue), anti-GAP45 (red), and either anti-RelB or anti-p52 (green). Scale bars = 10 μm. Plots display the nuclear-to-cytoplasmic intensity ratios of individual infected cells, with at least 300 cells analyzed per condition from at least three independent experiments. Black circles indicate the mean nuclear-to-cytoplasmic ratio of each independent biological replicate. Horizontal dashed lines indicate the overall mean. Statistical significance was determined using a one-way ANOVA with Tukey's multiple comparison test; ****$P < 0.0001$, ns = not significant.

Figure. S4

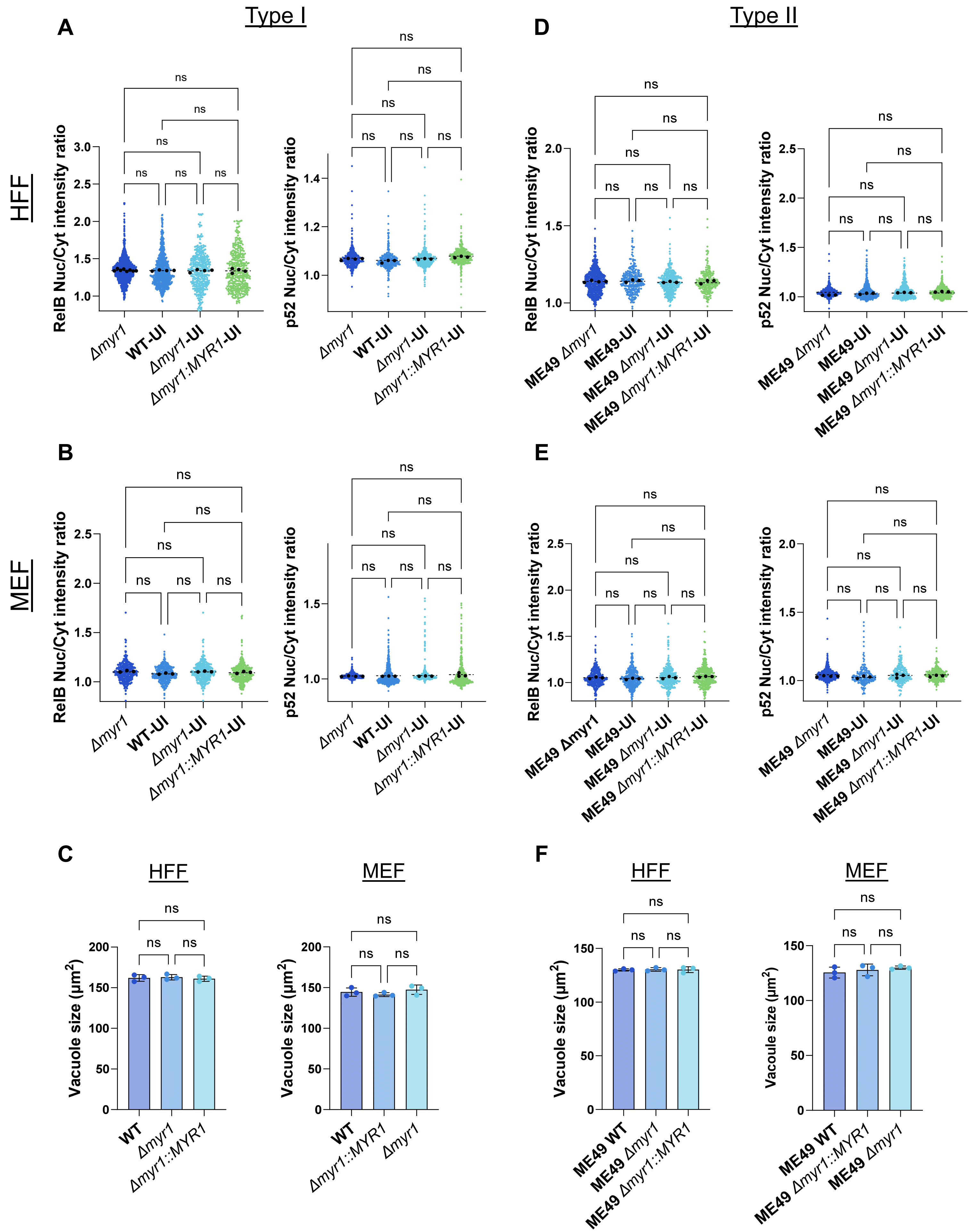
Type I
Type II
A
B
C
D
E
F
HFF
MEF
RelB Nuc/Cyt intensity ratio
p52 Nuc/Cyt intensity ratio
ns
Δmyr1
WT-UI
Δmyr1-UI
Δmyr1::MYR1-UI
ME49 Δmyr1
ME49-UI
ME49 Δmyr1-UI
ME49 Δmyr1::MYR1-UI
Vacuole size (μm²)
WT
Δmyr1::MYR1
ME49 WT
ME49 Δmyr1::MYR1

Figure S4. RelB and p52 nuclear-to-cytoplasmic ratios in uninfected bystander cells and parasitophorous vacuole size in HFF and MEF cells infected with Type I (RH) and Type II (ME49) *T. gondii* strains.

(**A-C**) HFF and MEF cells infected with WT, *Δmyr1*, or *Δmyr1::MYR1* complemented Type I (RH) parasites. (**D-F**) HFF and MEF cells infected with WT, *Δmyr1*, or *Δmyr1::MYR1* complemented Type II (ME49) parasites. (**A, B, D, E**) RelB and p52 Nuc/Cyt intensity ratios in uninfected HFF (**A, D**) and MEF (**B, E**) cells, compared to the RelB or p52 Nuc/Cyt ratio in *Δmyr1*-infected cells. (**C, F**) Average PV size. For nuclear-to-cytoplasmic ratio plots, at least 300 cells were analyzed per condition from at least three independent experiments. Black circles indicate the mean ratio of each independent biological replicate, and horizontal dashed lines indicate the overall mean. Vacuole size data are plotted as mean ± SD, with at least 300 vacuoles measured per condition from at least three independent experiments. Statistical significance was determined by one-way ANOVA with Tukey's multiple comparison test performed on the means of independent biological replicates. ns = not significant.

Figure. S5

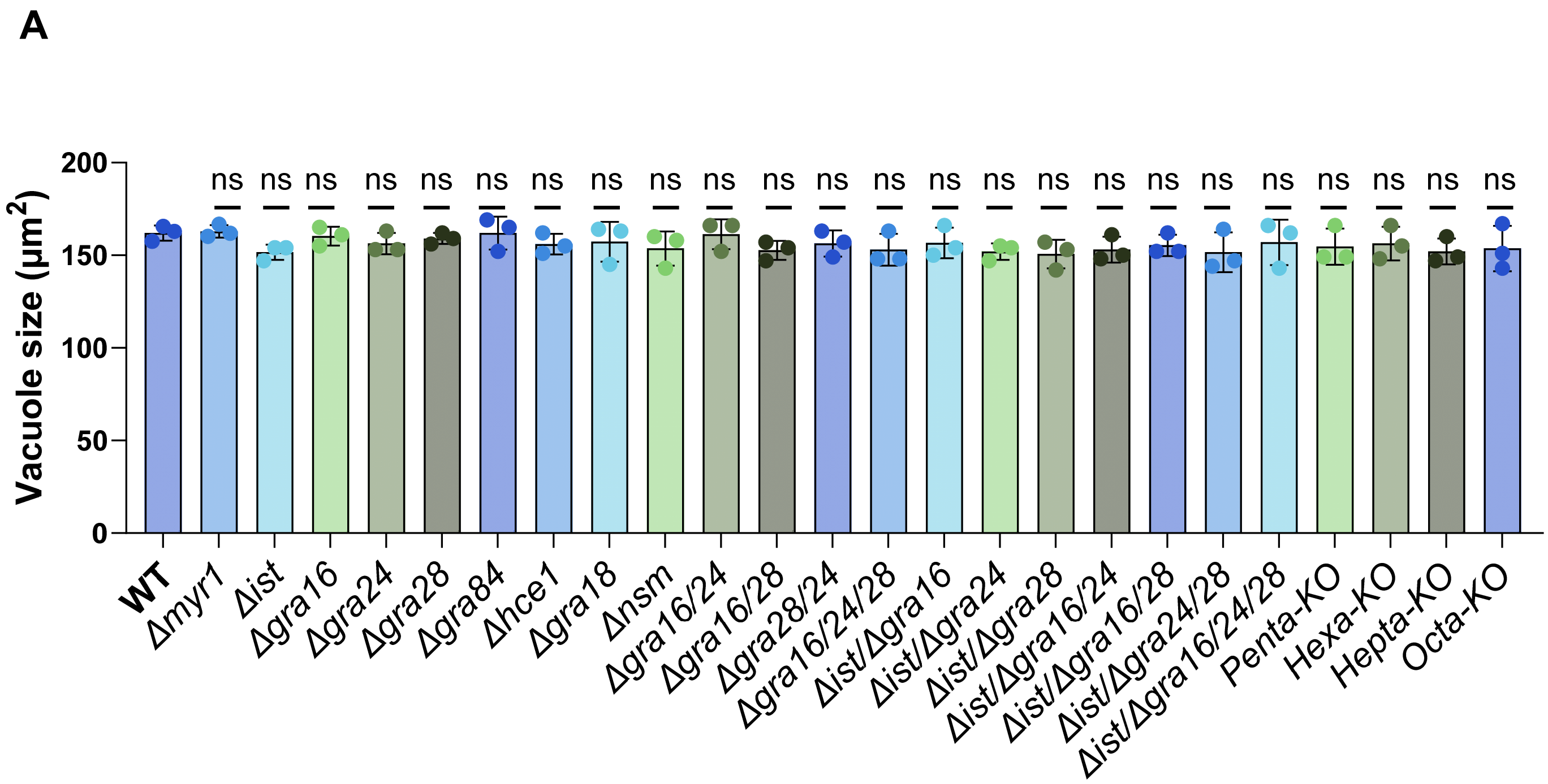
A
Vacuole size (µm²)
200
150
100
50
0
ns
WT
Δmyr1
Δist
Δgra16
Δgra24
Δgra28
Δgra84
Δhce1
Δgra18
Δnsm
Δgra16/24
Δgra16/28
Δgra28/24
Δgra16/24/28
Δist/Δgra16
Δist/Δgra24
Δist/Δgra28
Δist/Δgra16/24
Δist/Δgra16/28
Δist/Δgra24/28
Δist/Δgra16/24/28
Penta-KO
Hexa-KO
Hepta-KO
Octa-KO

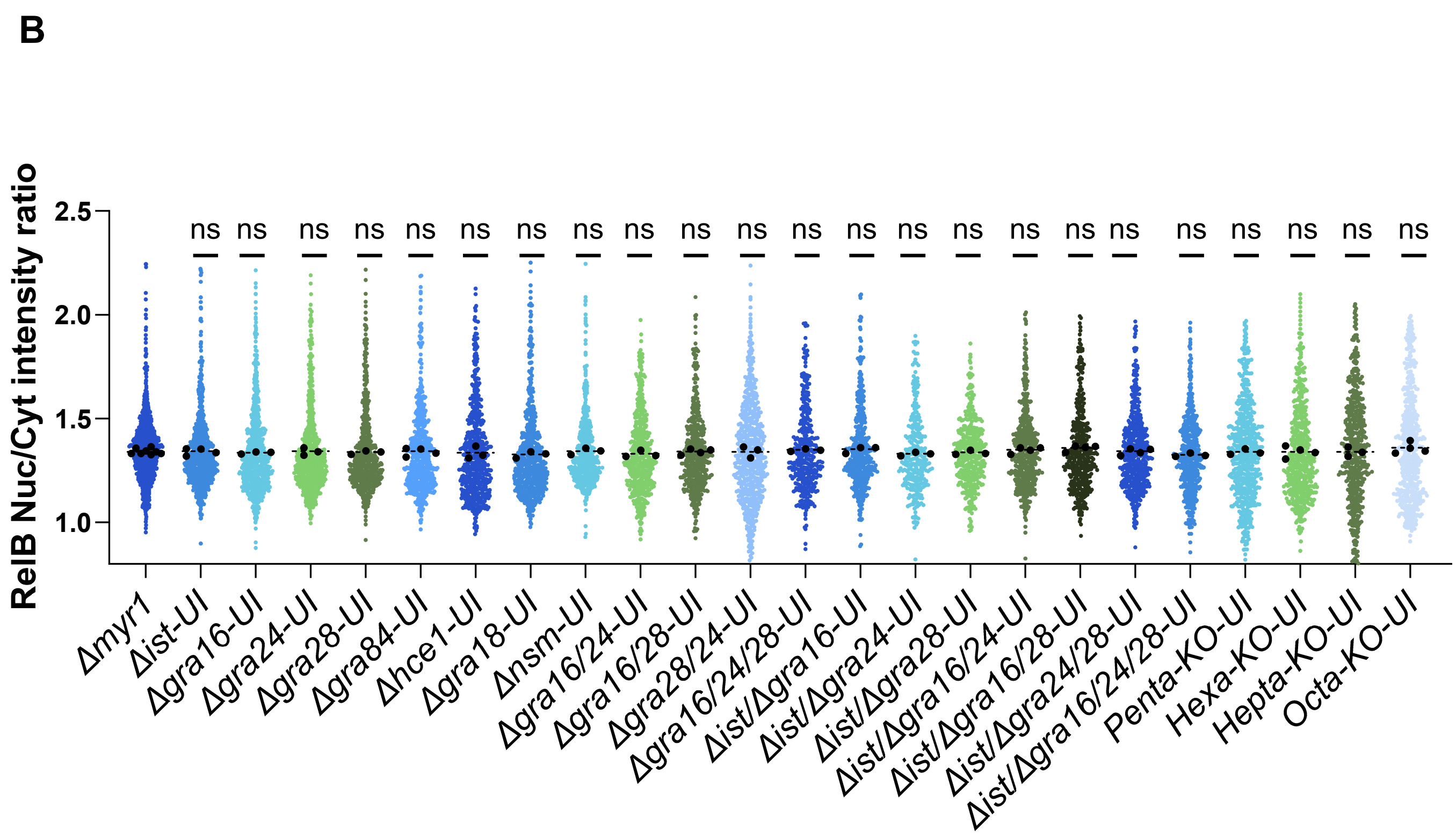
B
RelB Nuc/Cyt intensity ratio
2.5
2.0
1.5
1.0
ns
Δmyr1
Δist-UI
Δgra16-UI
Δgra24-UI
Δgra28-UI
Δgra84-UI
Δhce1-UI
Δgra18-UI
Δnsm-UI
Δgra16/24-UI
Δgra16/28-UI
Δgra28/24-UI
Δgra16/24/28-UI
Δist/Δgra16-UI
Δist/Δgra24-UI
Δist/Δgra28-UI
Δist/Δgra16/24-UI
Δist/Δgra16/28-UI
Δist/Δgra24/28-UI
Δist/Δgra16/24/28-UI
Penta-KO-UI
Hexa-KO-UI
Hepta-KO-UI
Octa-KO-UI

Figure S5. RelB nuclear-to-cytoplasmic ratio and vacuole size across single and combinatorial gene knockout RH strains.

(**A**) Average PV size in HFF cells infected with the indicated single and combinatorial knockout RH strains. (**B**) RelB Nuc/Cyt intensity ratio in uninfected HFF cells from fields infected with the indicated single and combinatorial knockout RH strains, compared to the RelB Nuc/Cyt ratio in *Δmyr1*-infected cells. For the Nuc/Cyt ratio plot, at least 300 cells were analyzed per condition from at least three independent experiments. Black circles indicate the mean ratio of each independent biological replicate, and horizontal dashed lines indicate the overall mean. Vacuole size data are plotted as mean ± SD, with at least 300 vacuoles measured per condition from at least three independent experiments. Statistical significance was determined by one-way ANOVA with Dunnett's multiple comparison test performed on the means of independent biological replicates. ns = not significant.

Figure. S6

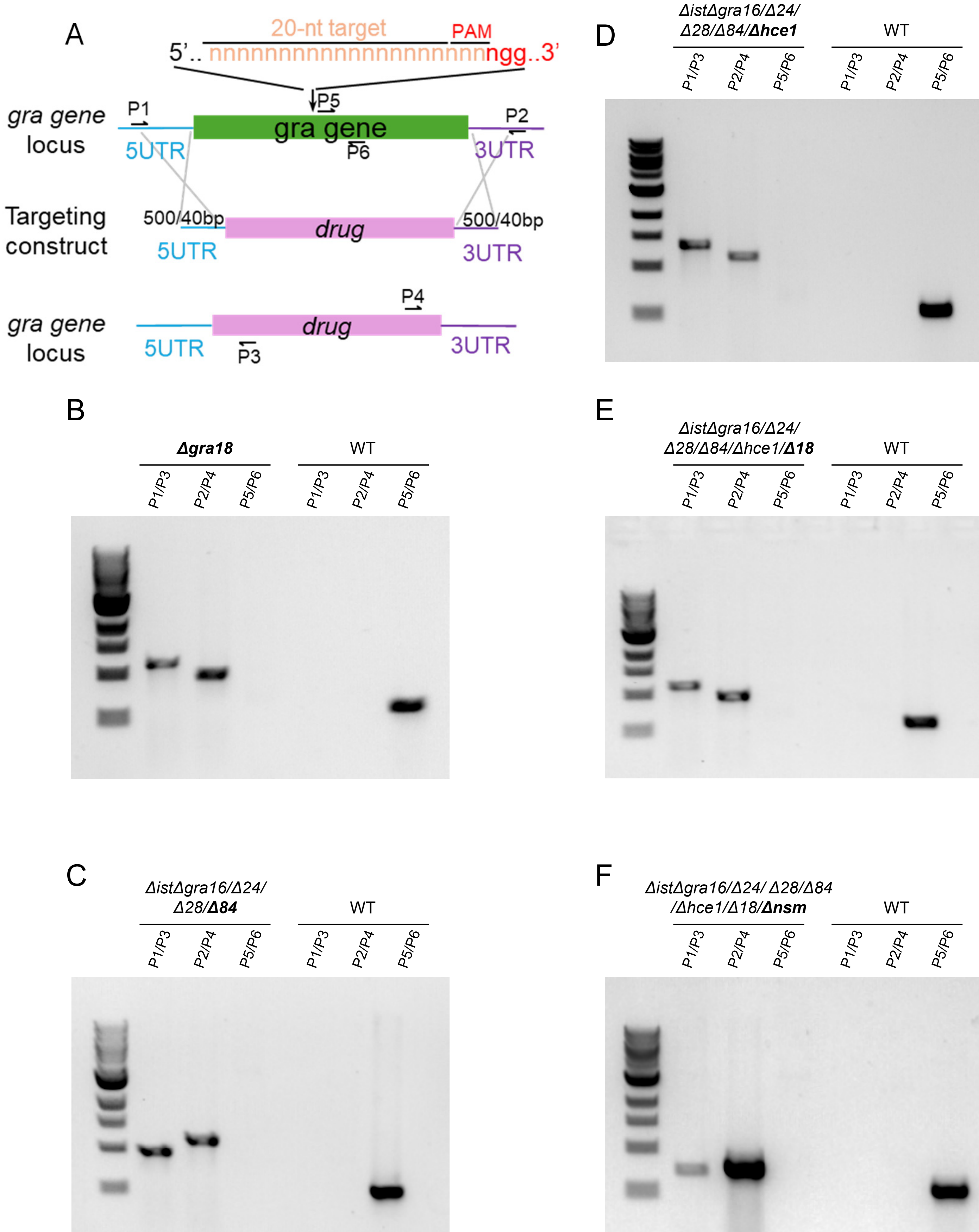

A
20-nt target
PAM
5’.. nnnnnnnnnnnnnnnnnnnnngg..3’
gra gene locus
P1
P5
P2
gra gene
5UTR
P6
3UTR
Targeting construct
500/40bp
drug
500/40bp
5UTR
3UTR
P4
gra gene locus
drug
5UTR
P3
3UTR
B
Δgra18
WT
P1/P3
P2/P4
P5/P6
P1/P3
P2/P4
P5/P6
C
ΔistΔgra16/Δ24/
Δ28/Δ84
WT
P1/P3
P2/P4
P5/P6
P1/P3
P2/P4
P5/P6
D
ΔistΔgra16/Δ24/
Δ28/Δ84/Δhce1
WT
P1/P3
P2/P4
P5/P6
P1/P3
P2/P4
P5/P6
E
ΔistΔgra16/Δ24/
Δ28/Δ84/Δhce1/Δ18
WT
P1/P3
P2/P4
P5/P6
P1/P3
P2/P4
P5/P6
F
ΔistΔgra16/Δ24/ Δ28/Δ84
/Δhce1/Δ18/Δnsm
WT
P1/P3
P2/P4
P5/P6
P1/P3
P2/P4
P5/P6

Figure S6. Generation and molecular validation of combinatorial knockout parasite strains.

(A) Schematic representation of the CRISPR/Cas9-mediated gene deletion strategy used to generate the transgenic strains in this study. sgRNA-expressing plasmids based on the pSAG1:CAS9-GFP, U6:sgUPRT backbone constitutively express a Cas9-GFP fusion and were used to target the middle of each gene, introducing a double-strand break to facilitate isolation of knockouts by homologous recombination. For single mutants, targeting constructs consisted of a selectable cassette flanked by short homology arms of approximately 40 bp positioned immediately upstream of the translation initiation site and downstream of the stop codon. Because available selectable markers were limiting for the quintuple through octuple knockout strains, donor amplicons containing approximately 500 bp homology arms flanking the selectable cassette were co-transfected with the Cas9-GFP plasmid, and the transiently expressed Cas9-GFP signal itself was used to sort live GFP-positive parasites for subcloning by limiting dilution, without drug selection.

(**B-F**) Diagnostic PCR verification of locus disruption for the indicated mutant strains: (B) Δgra18; (C) penta-KO, generated by addition of Δgra84 to the tetra-KO background (*Δist/Δgra16/Δgra24/Δgra28/Δgra84*); (D) hexa-KO, generated by addition of Δhce1 to the penta-KO background (*Δist/Δgra16/Δgra24/Δgra28/Δgra84/Δhce1*); (E) hepta-KO, generated by addition of Δgra18 to the hexa-KO background (*Δist/Δgra16/Δgra24/Δgra28/Δgra84/Δhce1/Δgra18*); and (F) octa-KO, generated by addition of Δnsm to the hepta-KO background (*Δist/Δgra16/Δgra24/Δgra28/Δgra84/Δhce1/Δgra18/Δnsm*). Primer binding sites are

indicated in (**A**): P1/P3 and P2/P4 confirm integration of the left and right homology arms, respectively; P5/P6 examines the integrity of the endogenous locus. Successful knockout clones yielded PCR products with P1/P3 and P2/P4 but no product with P5/P6, whereas wild-type parasites showed the opposite pattern. See Table S3 for oligonucleotide sequences.

Figure. S7

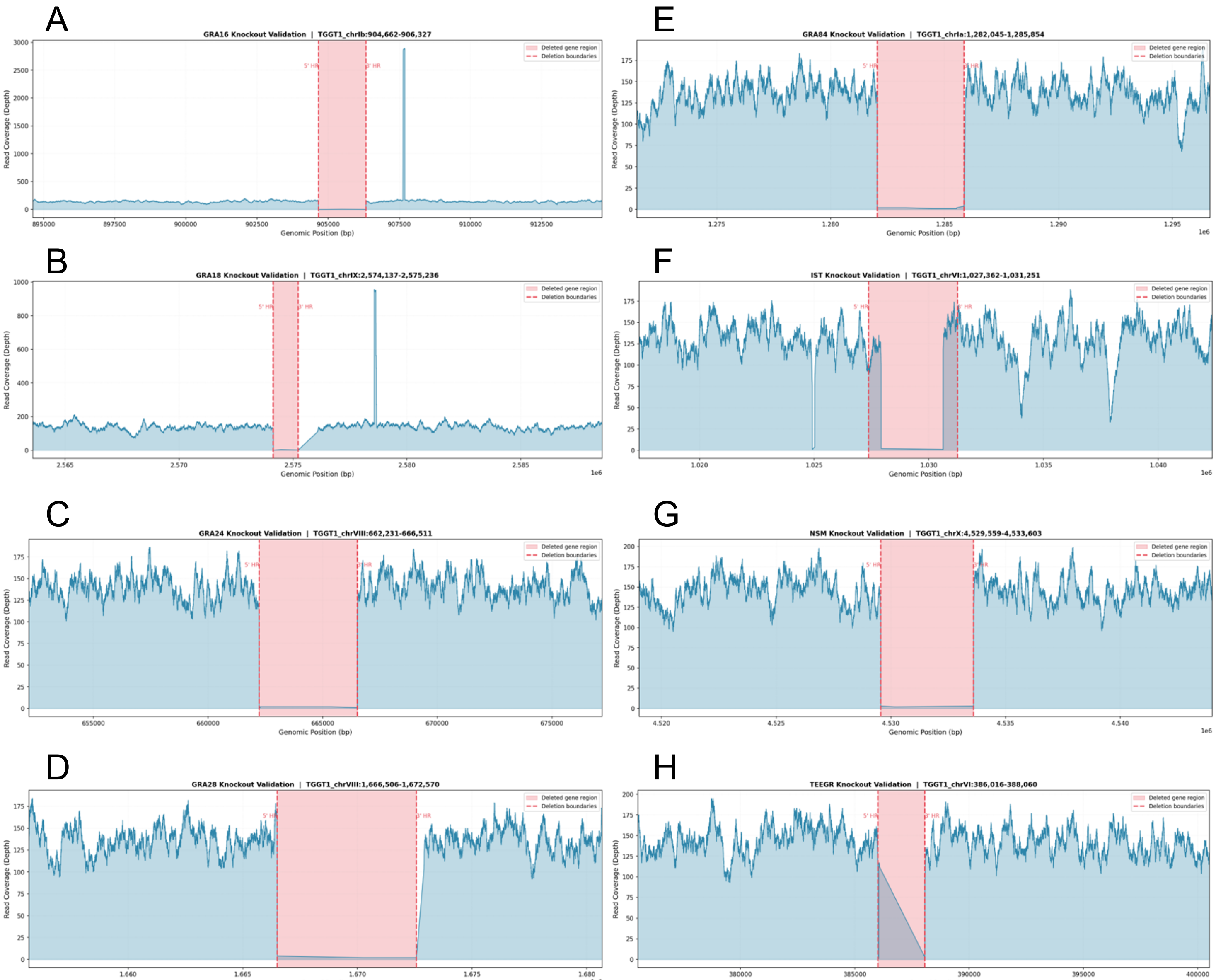
A
GRA16 Knockout Validation | TGGT1_chrIb:904,662-906,327
B
GRA18 Knockout Validation | TGGT1_chrIX:2,574,137-2,575,236
C
GRA24 Knockout Validation | TGGT1_chrVIII:662,231-666,511
D
GRA28 Knockout Validation | TGGT1_chrVIII:1,666,506-1,672,570
E
GRA84 Knockout Validation | TGGT1_chrIa:1,282,045-1,285,854
F
IST Knockout Validation | TGGT1_chrVI:1,027,362-1,031,251
G
NSM Knockout Validation | TGGT1_chrX:4,529,559-4,533,603
H
TEEGR Knockout Validation | TGGT1_chrVI:386,016-388,060
Deleted gene region
Deletion boundaries
5' HR
3' HR
Read Coverage (Depth)
Genomic Position (bp)

Figure S7. Whole genome sequencing read coverage across the eight target loci deleted in the octa-KO strain.

(**A-H**) Whole genome sequencing read coverage across each of the eight target loci deleted in the octa-KO strain: GRA16 (**A**), GRA18 (**B**), GRA24 (**C**), GRA28 (D), GRA84 (**E**), IST (**F**), NSM (**G**), and TEEGR/HCE1 (**H**). Read coverage depth is plotted across genomic position for each locus. Shaded regions indicate the deleted gene boundaries as defined by the CRISPR targeting strategy and dashed lines mark the deletion boundaries.
