# Supplementary material for: A suite of eight Toxoplasma gondii effectors cooperates to activate the non-canonical NF-κB pathway
Source: mBio. 2026 Aug 28;17(10):e01652-26. doi: 10.1128/mbio.01652-26 (PMC13712946; doi:10.1128/mbio.01652-26)
Supplement: Supplementary file 2 [file mbio.01652-26-s0002.docx]

**Table S1 - *Toxoplasma gondii* strains used in this study**

| **Line** | **Genotype** | **Source** |
| --- | --- | --- |
| RH (WT) | RH*ΔhxgprtΔku80* | (Huynh and Carruthers, 2009) |
| RH *Δmyr1* | RH*Δhxgprt Δmyr1::HX* | (Franco et al., 2016) |
| RH *Δmyr1/MYR1* | RH*Δhxgprt*  *Δmyr1/MYR1::HX* | (Franco et al., 2016) |
| RH *Δist* | RH*ΔhxgprtΔku80*; *Δist::DHFR-TS* | (Kongsomboonvech et al., 2023) |
| RH *Δnsm* | RH*ΔhxgprtΔku80*; *Δnsm::HX* | (Kongsomboonvech et al., 2023) |
| RH *Δgra16* | RH*ΔhxgprtΔku80*; *Δgra16::CAT* | (Henry et al., 2024) |
| RH *Δgra18* | RH*ΔhxgprtΔku80; Δgra18::*DHFR-TS | This study |
| RH *Δgra24* | RH*ΔhxgprtΔku80*; *Δgra24::CAT* | (Henry et al., 2024) |
| RH *Δgra28* | RH*ΔhxgprtΔku80*; *Δgra28::HX* | (Henry et al., 2024) |
| RH *Δgra84* | RH*ΔhxgprtΔku80*; *Δgra84::HX* | (Thind et al., 2025) |
| RH *Δhce1* | RH*ΔhxgprtΔku80*; *Δhce1::DHFR-TS* | (Pierre-Louis et al., 2022) |
| RH *Δist,Δgra16* | RH*ΔhxgprtΔku80*; *Δist::DHFR-TS, Δgra16::CAT* | (Henry et al., 2024) |
| RH *Δist,Δgra24* | RH*ΔhxgprtΔku80*; *Δist::DHFR-TS, Δgra24::CAT* | (Henry et al., 2024) |
| RH *Δist,Δgra28* | RH*ΔhxgprtΔku80*; *Δist::DHFR-TS, Δgra28::HX* | (Henry et al., 2024) |
| RH *Δgra16,Δgra24* | RH*ΔhxgprtΔku80*; *Δgra16::CAT, Δgra24::PHLEO* | (Henry et al., 2024) |
| RH *Δgra16,Δgra28* | RH*ΔhxgprtΔku80*; *Δgra16::CAT, Δgra28::HX* | (Henry et al., 2024) |
| RH *Δgra24,Δgra28* | RH*ΔhxgprtΔku80*; *Δgra24::CAT, Δgra28::HX* | (Henry et al., 2024) |
| RH *Δist,Δgra16,Δgra24* | RH*ΔhxgprtΔku80*; *Δist::DHFR-TS, Δgra16::CAT, Δgra24::PHLEO* | (Henry et al., 2024) |
| RH *Δist,Δgra16,Δgra28* | RH*ΔhxgprtΔku80*; *Δist::DHFR-TS, Δgra16::CAT, Δgra28::HX* | (Henry et al., 2024) |
| RH *Δist,Δgra24,Δgra28* | RH*ΔhxgprtΔku80*; *Δist::DHFR-TS, Δgra24::CAT, Δgra28::HX* | (Henry et al., 2024) |
| RH *Δgra16,Δgra24,Δgra28* | RH*ΔhxgprtΔku80*; *Δgra16::CAT, Δgra24::PHLEO, Δgra28::HX* | (Henry et al., 2024) |
| RH *Δist,Δgra16,Δgra24,Δgra28* | RH*ΔhxgprtΔku80*; *Δist::DHFR-TS, Δgra16::CAT, Δgra24::PHLEO, Δgra28::HX* | (Henry et al., 2024) |
| RH *Δist,Δgra16,Δgra24,Δgra28,∆gra84* | RH*ΔhxgprtΔku80*; *Δist::DHFR-TS, Δgra16::CAT, Δgra24::PHLEO, Δgra28::HX, Δgra84::HX* | This study |
| RH *Δist,Δgra16,Δgra24,Δgra28,∆gra84,∆hce1* | RH*ΔhxgprtΔku80*; *Δist::DHFR-TS, Δgra16::CAT, Δgra24::PHLEO, Δgra28::HX, Δgra84::HX, Δhce1::DHFR-TS* | This study |
| RH *Δist,Δgra16,Δgra24,Δgra28,∆gra84,∆hce1,∆gra18* | RH*ΔhxgprtΔku80*; *Δist::DHFR-TS, Δgra16::CAT, Δgra24::PHLEO, Δgra28::HX, Δgra84::HX, Δhce1::DHFR-TS, Δgra18::*DHFR-TS | This study |
| RH *Δist,Δgra16,Δgra24,Δgra28,∆gra84,∆hce1,∆gra18,∆nsm* | RH*ΔhxgprtΔku80*; *Δist::DHFR-TS, Δgra16::CAT, Δgra24::PHLEO, Δgra28::HX, Δgra84::HX, Δhce1::DHFR-TS, Δgra18::*DHFR-TS, *Δnsm::HX* | This study |
| ME49 (WT) | ME49 *Δhxgprt::FLUC* | (Tobin and Knoll, 2012) |
| ME49 *Δmyr1* | ME49 Δhxgprt::FLUC; *Δmyr1::HX* | (Franco et al., 2016) |
| ME49 *Δmyr1/MYR1* | ME49 Δhxgprt::FLUC; *Δmyr1/MYR1::HX* | (Franco et al., 2016) |

**References:**

Franco, M., Panas, M.W., Marino, N.D., Lee, M.C., Buchholz, K.R., Kelly, F.D., Bednarski, J.J., Sleckman, B.P., Pourmand, N., and Boothroyd, J.C. (2016). A Novel Secreted Protein, MYR1, Is Central to Toxoplasma's Manipulation of Host Cells. mBio *7*, e02231-02215.

Henry, B., Phillips, A.J., Sibley, L.D., and Rosenberg, A. (2024). A combination of four Toxoplasma gondii nuclear-targeted effectors protects against interferon gamma-driven human host cell death. mBio *15*, e0212424.

Huynh, M.H., and Carruthers, V.B. (2009). Tagging of endogenous genes in a Toxoplasma gondii strain lacking Ku80. Eukaryot Cell *8*, 530-539.

Kongsomboonvech, A.K., Garcia-Lopez, L., Njume, F., Rodriguez, F., Souza, S.P., Rosenberg, A., and Jensen, K.D.C. (2023). Variation in CD8 T cell IFNgamma differentiation to strains of Toxoplasma gondii is characterized by small effect QTLs with contribution from ROP16. Front Cell Infect Microbiol *13*, 1130965.

Pierre-Louis, E., Etheridge, M.G., de Paula Baptista, R., Khan, A., Chasen, N.M., and Etheridge, R.D. (2022). Disruption of Toxoplasma gondii-Induced Host Cell DNA Replication Is Dependent on Contact Inhibition and Host Cell Type. mSphere *7*, e0016022.

Thind, A.C., Mead, A.J., Quan, J.J., Sha, J., Wohlschlegel, J.A., and Bradley, P.J. (2025). Proteolytic processing and host nuclear targeting of the novel effector GRA84 in Toxoplasma gondii. Mol Biol Cell *36*, ar97.

Tobin, C.M., and Knoll, L.J. (2012). A patatin-like protein protects Toxoplasma gondii from degradation in a nitric oxide-dependent manner. Infect Immun *80*, 55-61.
