# Supplementary material for: A suite of eight Toxoplasma gondii effectors cooperates to activate the non-canonical NF-κB pathway
Source: mBio. 2026 Aug 28;17(10):e01652-26. doi: 10.1128/mbio.01652-26 (PMC13712946; doi:10.1128/mbio.01652-26)
Supplement: Supplementary file 3 [file mbio.01652-26-s0003.docx]

**Table S2. Plasmids used in this study.**

| **Common Name** | **Description** | **Source** |
| --- | --- | --- |
| pSAG1:CAS9-GFP, U6:sgUPRT | Template for making GOI targeting Cas9 plasmids | Addgene (#54467)  (Shen et al., 2014) |
| pSAG1:CAS9-GFP, U6:sgGRA18 | CRISPR/CAS9 plasmid targeting GRA18 coding sequence for gene deletion | This study |
| pSAG1:CAS9-GFP, U6:sgGRA84 | CRISPR/CAS9 plasmid targeting GRA84 coding sequence for gene deletion | This study |
| pSAG1:CAS9-GFP, U6:sgHCE1 | CRISPR/CAS9 plasmid targeting HCE1 coding sequence for gene deletion | This study |
| pSAG1:CAS9-GFP, U6:sgNSM | CRISPR/CAS9 plasmid targeting NSM coding sequence for gene deletion | (Rosenberg and Sibley, 2021) |
| pFloxed DHFR-TS* | Template for amplification of DHFR resistance cassette for obtaining deletion strains | In-house |
| pLinker-2xTy-HXGPRT-LoxP | Template for amplification of HX resistance cassette for obtaining deletion strains | In-house |

**References:**

Rosenberg, A., and Sibley, L.D. (2021). Toxoplasma gondii secreted effectors co-opt host repressor complexes to inhibit necroptosis. Cell Host Microbe *29*, 1186-1198 e1188.

Shen, B., Brown, K.M., Lee, T.D., and Sibley, L.D. (2014). Efficient gene disruption in diverse strains of Toxoplasma gondii using CRISPR/CAS9. mBio *5*, e01114-01114.
